# Gating of hair cell Ca^2+^ channels governs the activity of cochlear neurons

**DOI:** 10.1126/sciadv.adu7898

**Published:** 2025-06-18

**Authors:** Nare Karagulyan, Anupriya Thirumalai, Susann Michanski, Yumeng Qi, Qinghua Fang, Haoyu Wang, Nadine J. Ortner, Jörg Striessnig, Nicola Strenzke, Carolin Wichmann, Yunfeng Hua, Tobias Moser

**Affiliations:** ^1^ Institute for Auditory Neuroscience and InnerEarLab, University Medical Center Göttingen, Göttingen, Germany.; ^2^ Auditory Neuroscience & Synaptic Nanophysiology Group, Max Planck Institute for Multidisciplinary Sciences, Göttingen, Germany.; ^3^ Göttingen Graduate School for Neurosciences and Molecular Biosciences, University of Göttingen, Göttingen, Germany.; ^4^ Cluster of Excellence “Multiscale Bioimaging: from Molecular Machines to Networks of Excitable Cells” (MBExC), University of Göttingen, Göttingen, Germany.; ^5^ Center for Biostructural Imaging of Neurodegeneration (BIN), University Medical Center Göttingen, Göttingen, Germany.; ^6^ Shanghai Institute of Precision Medicine, Shanghai Ninth People’s Hospital, Shanghai Jiao Tong University School of Medicine, Shanghai, China.; ^7^ Department of Pharmacology and Toxicology, Center for Molecular Biosciences Innsbruck, University of Innsbruck, Innsbruck, Austria.; ^8^ Auditory Systems Physiology Group, Institute for Auditory Neuroscience and InnerEarLab, University Medical Center Göttingen, Göttingen, Germany.; ^9^ Molecular Architecture of Synapses Group, Institute for Auditory Neuroscience and InnerEarLab, University Medical Center Göttingen, Göttingen, Germany.; ^10^ Department for Hearing, Speech and Voice Disorders, Medical University of Innsbruck, 6020 Innsbruck, Austria.

## Abstract

Our sense of hearing processes sound intensities spanning six orders of magnitude. In the ear, the receptor potential of presynaptic inner hair cells (IHCs) covers the entire intensity range, while postsynaptic spiral ganglion neurons (SGNs) tile the range with their firing rate codes. IHCs vary the voltage dependence of Ca^2+^ channel activation among their active zones (AZs), potentially diversifying SGN firing. Here, we tested this hypothesis in mice modeling the human Ca_V_1.3^A749G^ mutation that causes low-voltage Ca^2+^ channel activation. We demonstrate activation of Ca^2+^ influx and glutamate release of IHC AZs at lower voltages, increased spontaneous firing in SGNs, and lower sound threshold of Ca_V_1.3^A749G/A749G^ mice. Loss of synaptic ribbons in IHCs at ambient sound levels of mouse husbandry indicates that low-voltage Ca^2+^ channel activation poses a risk for noise-induced synaptic damage. We propose that the heterogeneous voltage dependence of Ca_V_1.3 activation among presynaptic IHC AZs contributes to the diversity of firing among the postsynaptic SGNs.

## Teaser

Voltage dependence of presynaptic Ca^2+^ channel activation impacts postsynaptic firing and shapes synaptic vulnerability.

## INTRODUCTION

Inner hair cells (IHCs) sample sound information at each tonotopic position of the cochlea and convey it to spiral ganglion neurons (SGNs) via afferent ribbon synapses that vary in structure and function (*1*–*3*). For example, while all IHC glutamate release depends on Ca^2+^ influx through Ca_V_1.3 channels (*4*, *5*) that are affected by human mutations in *CACNA1D* (*6*, *7*), IHCs vary the voltage dependence of Ca_V_1.3 Ca^2+^ influx among their active zones (AZs) (*8*, *9*). This AZ heterogeneity might contribute to diverse SGN responses that enable a tiling of the audible range of sound pressures by the rate codes of the individual SGNs.

This collective intensity coding by functionally diverse SGNs likely supports high-fidelity processing across the 120-dB range (six orders of magnitude) of audible sound pressures (*1*). Aside from the different fractions of sound intensity range covered by changes in their firing rate, SGNs also vary in their spontaneous firing rate (SR): from negligible firing to SR > 100 spikes/s (*10*–*13*). SR results from glutamate release triggered by Ca^2+^ influx through L-type voltage-gated Ca^2+^ channels (*14*) at the IHC resting membrane potential (*15*) that is governed by the balance of the baseline mechanotransducer current and voltage-gated K^+^ currents (*16*).

A current challenge is to causally relate synaptic heterogeneity (*9*, *17*–*23*) to the physiology (*10*, *12*, *24*–*27*) of SGNs that also show diverse molecular profiles (*28*–*31*). For the interpretation of findings regarding SGN function, the field has adopted the concept of a spatial segregation of SGN synapses on the basal pole of the IHC. SGNs with low SR and high sound threshold (“low-SR”) preferentially synapse on the “modiolar” IHC side (facing the modiolus, i.e., cochlear center), while high-SR, low-threshold (“high-SR”) SGNs tend to innervate the opposite “pillar” side of the IHC (facing the pillar cells) (*20*, *32*). For example, the molecularly defined type I_b_ and I_c_ SGNs tend to innervate the modiolar side and, hence, have been considered to represent low-SR SGNs, e.g., due to different excitability (*27*–*29*, *33*, *34*).

Similarly, the finding that pillar synapses activate at lower voltages than modiolar ones (*17*, *18*, *35*) has sparked the hypothesis that differences in the voltage range of synapse operation contribute to the variance in spontaneous and sound-evoked firing among SGNs at a given tonotopic position. Pillar AZs with lower activation voltage of Ca_V_1.3 channels and tighter spatial coupling to synaptic vesicles (SVs) have higher rates of glutamate release at the IHC resting potential and lower thresholds for evoked release (*35*). This predicts higher SR and lower sound thresholds of their postsynaptic SGNs compared to the modiolar AZs that preferentially connect to SGNs with lower SR. A previous study showed the activation of IHC Ca^2+^ influx at lower voltages and concomitantly increased SR in SGNs of deaf mouse mutants for *Gipc3*, a putative Ca_V_1.3 regulator (*17*). However, further testing of this presynaptic hypothesis is required, ideally combining synaptic and system physiology with computational modeling in mice with altered Ca^2+^ channel gating but preserved hearing.

Here, we capitalized on Ca_V_1.3^A749G^ (or *Cacna1d* ^A749G^) mouse mutants (*36*) modeling the human *CACNA1D* p.A749G mutation (*7*). While the loss of Ca_V_1.3 function causes syndromic deafness (*6*), the *CACNA1D* p.A749G mutation belongs to a group of mutations that cause aberrant gating and often are associated with neurodevelopmental disorders with and without endocrine symptoms but so far uncertain hearing function (*37*, *38*).

The *CACNA1D* p.A749G mutation shifts the Ca_V_1.3 activation to lower voltages by which Ca_V_1.3^A749G^ mice could provide an opportunity to test the presynaptic hypothesis of functional SGN diversity. However, as it also shifts inactivation to lower voltages (*7*), the Ca_V_1.3^A749G^ mutation might also reduce the number of activatable Ca^2+^ channels of IHCs that are thought to rest at a potential −58 mV in the absence of sound stimulation in vivo (*16*). Enhanced Ca_V_1.3 inactivation due to lack of Ca^2+^ binding protein(s) 1 and 2 impairs hearing by reducing the number of activatable Ca_V_1.3 channels mediating synaptic sound encoding (*39*, *40*), with *CABP2* being another deafness gene (*41*).

Hence, it was conceivable that hetero- and homozygous Ca_V_1.3^A749G^ mice (for simplicity, we nicknamed the mutant allele Ca_V_1.3^AG^) and the (heterozygous) Ca_V_1.3^AG^ patients could either show supernatural acoustic sensitivity or, in case of excessive Ca_V_1.3 inactivation, be hearing impaired. Last, Ca_V_1.3 gain of function might lead to noise-induced synaptic damage or synaptopathy (*42*–*44*) even at otherwise nondamaging noise levels.

Here, we combined physiological and morphological approaches to characterize the auditory system of Ca_V_1.3^AG/AG^ and Ca_V_1.3^AG/WT^ mice. We integrate our findings by computational modeling, demonstrate the impact of the lower voltage of Ca_V_1.3 activation on synaptic structure and function, and support the presynaptic hypothesis of functional SGN diversity.

## RESULTS

### A749G mutation in *Cacna1D* causes a hyperpolarized shift of synapse activation

**Fig. 1.**
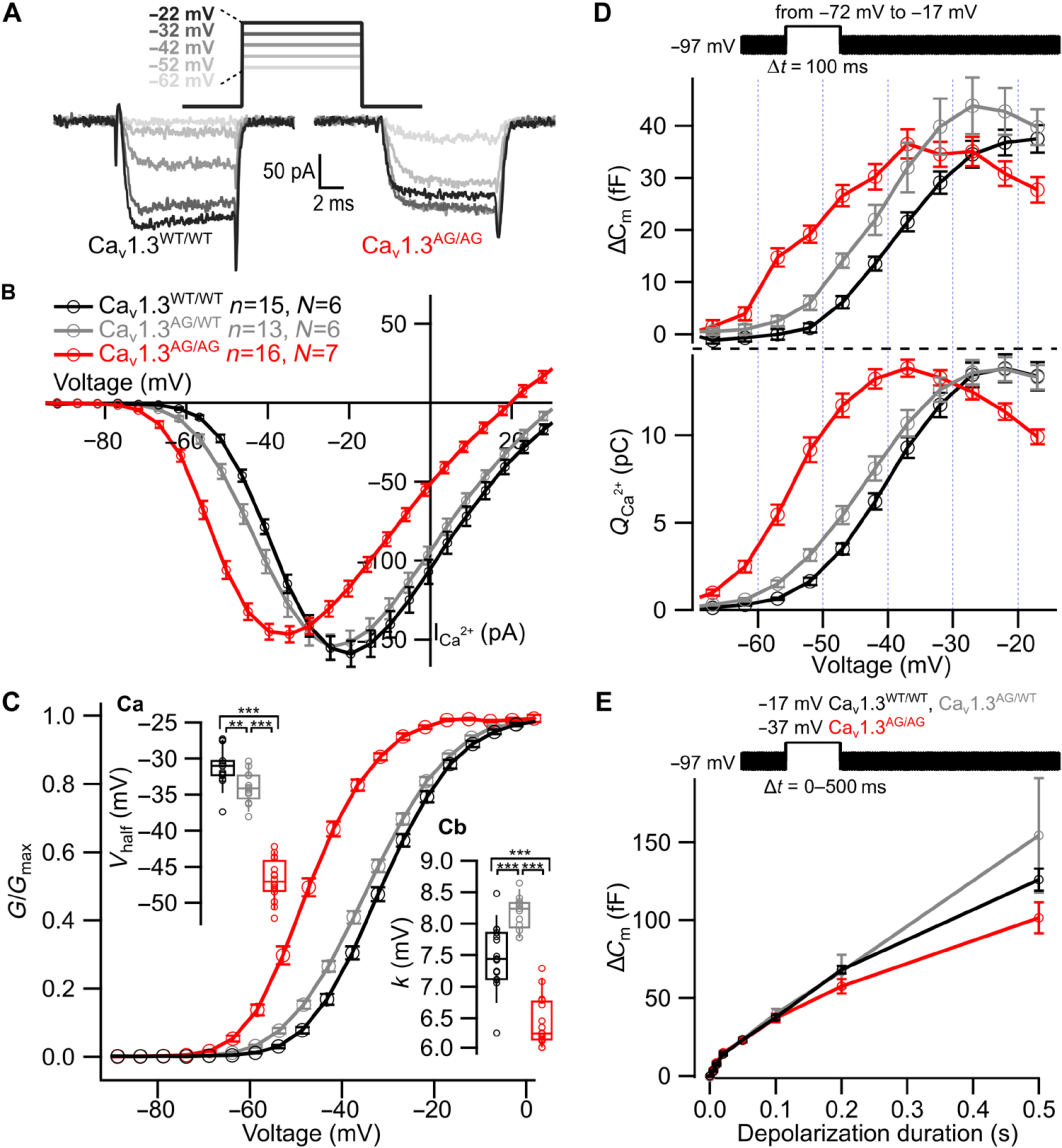
Shift to lower voltages and altered voltage sensitivity of Ca_V_1.3 activation in IHCs of Ca_V_1.3^AG/WT^ and Ca_V_1.3^AG/AG^ mice. (**A**) Representative Ca^2+^ current traces from Ca_V_1.3^WT/WT^ (bottom left) and Ca_V_1.3^AG/AG^ (bottom right) IHCs evoked by step depolarizations (top). (**B**) Whole-cell Ca^2+^ current-voltage relationships (*I*-*V* curves) show comparable maximal Ca^2+^ current amplitude in Ca_V_1.3^WT/WT^, Ca_V_1.3^AG/WT^, and Ca_V_1.3^AG/AG^ IHCs. Error bars show ± SEM. (**C**) Ca^2+^ channel activation–voltage relationships calculated from *I*-*V* curves show a hyperpolarized shift in Ca_V_1.3^AG/WT^ and Ca_V_1.3^AG/AG^ IHCs. Error bars show ± SEM. (Ca) The voltage of half-maximal activation (*V*_half_) is hyperpolarized in Ca_V_1.3^AG/WT^ and Ca_V_1.3^AG/AG^ IHCs. (Cb) The voltage sensitivity (*k*) is decreased in Ca_V_1.3^AG/WT^ and increased in Ca_V_1.3^AG/AG^ IHCs compared to the controls. (**D**) Mean exocytic change in membrane capacitance (Δ*C*_m_) and Ca^2+^ current integrals (*Q*_Ca_) evoked by 100-ms pulses of different depolarizations. (**E**) Mean exocytic Δ*C*_m_ in response to different depolarization durations. Data in (B) to (D) are presented as mean ± SEM. Box-whisker plots with individual data points overlaid show the median, 25th, and 75th percentiles (box) and 10th and 90th percentiles (whiskers). Statistical significances were determined using one-way analysis of variance (ANOVA), followed by Tukey’s post hoc test for (Ca) and (Cb). Significances are reported as **P < 0.01 and ***P < 0.001.

To characterize the impact of the A749G mutation on IHC Ca^2+^ influx, we first analyzed voltage-gated Ca^2+^ currents of IHCs at the whole-cell and synaptic levels in apical cochlear coils, acutely dissected from hearing mice [postnatal days (p) 21 to 28]. Maximal Ca^2+^ current amplitudes of Ca_V_1.3^AG/WT^ and Ca_V_1.3^AG/AG^ IHCs (Fig. 1, A and B) were not significantly different from control (Ca_V_1.3^WT/WT^), similar to what was observed in adrenal chromaffin cells of Ca_V_1.3^AG/WT^ mice (*36*), but contrasting the increased amplitudes found upon heterologous expression of the mutant Ca_V_1.3 channel (*7*). However, in keeping with heterologous expression (*7*), we found a gene dose–dependent shift of Ca^2+^ channel activation to lower voltages and increased voltage sensitivity of activation in Ca_V_1.3^AG/AG^ IHCs (Fig. 1, Ca and Cb).

Alike Ca^2+^ influx, exocytosis of Ca_V_1.3^AG/AG^ assessed as change in membrane capacitance (Δ*C*_m_) was activated at lower voltages (Fig. 1D). When adjusting depolarizations to the potential of maximal Ca^2+^ influx in Ca_V_1.3^AG/AG^ IHCs (−37 mV versus –17 mV in Ca_V_1.3^WT/WT^), we found IHC exocytosis largely unaltered (Fig. 1E). The kinetics of activation and inactivation of Ca^2+^ channels in IHCs of Ca_V_1.3^AG/WT^ and Ca_V_1.3^AG/AG^ mice were unaltered, but as found with heterologous expression (*7*), deactivation was slowed significantly in IHCs of both genotypes, suggesting a higher prevalence of longer open times [mode 2 (*45*); fig. S1]. Nonstationary fluctuation analysis of IHC Ca^2+^ influx (*46*, *47*) revealed an increase in the open probability, which was balanced by a decrease in the activatable number of Ca^2+^ channels in Ca_V_1.3^AG/AG^ IHCs (fig. S2).

Next, we tested whether this decrease reflects steady-state inactivation or a potential homeostatic reduction of the Ca_V_1.3 channels. Performing immunohistochemistry, we revealed a reduced number of Ca_V_1.3 channels at AZs of Ca_V_1.3^AG/AG^ IHCs evident as lower Ca_V_1.3 immunofluorescence intensity in confocal microscopy (fig. S3, A and C) and reduced size of Ca_V_1.3 channel clusters in stimulated emission depletion (STED) nanoscopy (fig. S3, D and E). Likewise, the Ribeye/Ctbp2 immunofluorescence intensity was reduced, indicating a concomitant decrease in ribbon size in apical IHCs (fig. S3B).

**Fig. 2.**
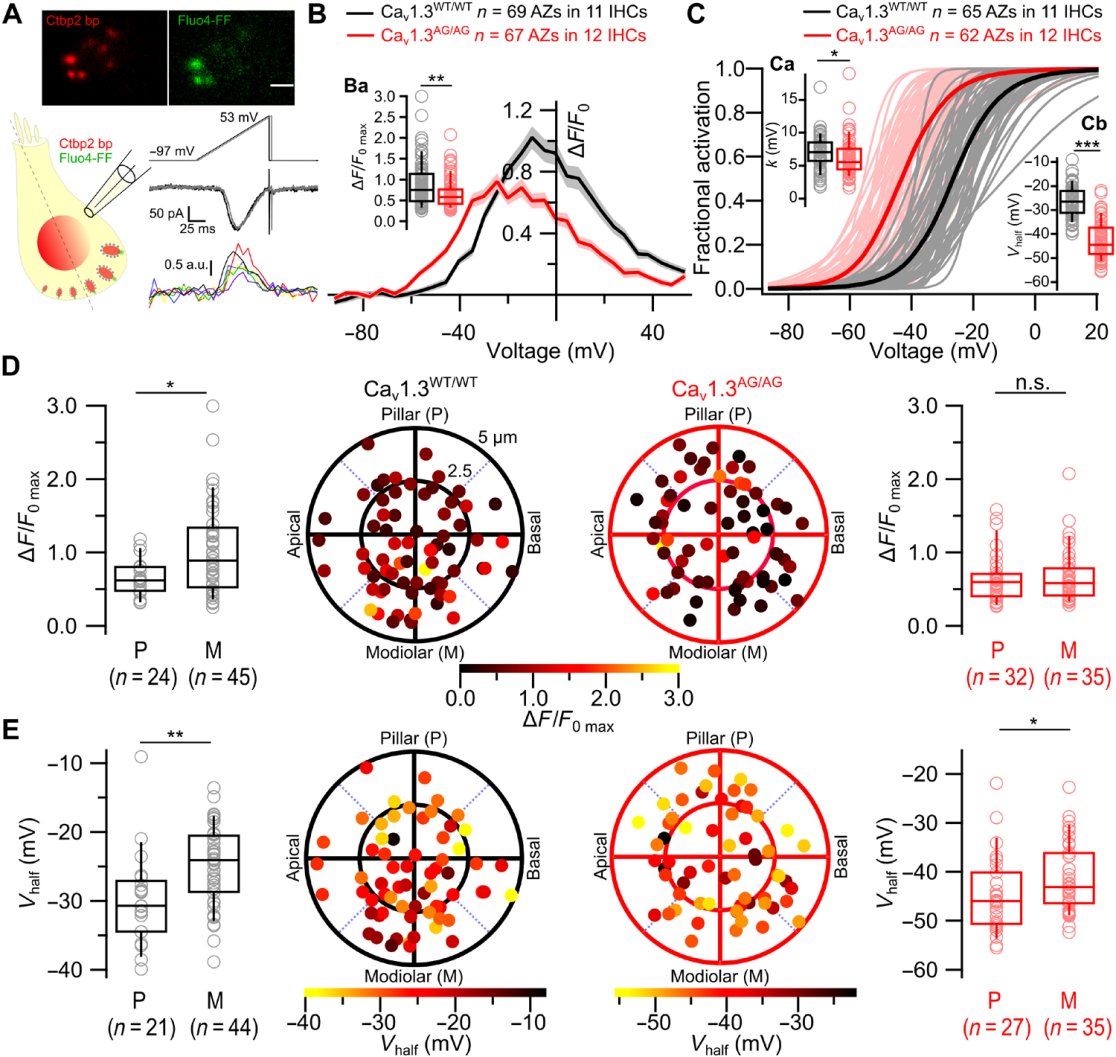
Reduced amplitude, hyperpolarized activation, and altered voltage sensitivity of synaptic Ca^2+^ influx in Ca_V_1.3^AG/AG^ IHCs. (**A**) Single confocal plane of a representative Ca_V_1.3^WT/WT^ IHC showing TAMRA-conjugated dimeric Ribeye/Ctbp2 peptide and Fluo4-FF fluorescence. Scale bar, 2 μm. Black and gray colors of voltage ramp stimuli and corresponding whole-cell Ca^2+^ currents show IHC response to two voltage ramp depolarizations. Intensity-time profiles of single Ca^2+^ hotspots from one IHC are shown with different colors. bp, binding peptide; a.u., arbitrary units. (**B**) Average fluorescence-voltage (*F*-*V*) relationships of Ca^2+^ influx at single AZs of Ca_V_1.3^WT/WT^ and Ca_V_1.3^AG/AG^ IHCs. Shaded areas show ± SEM. (Ba) The maximal Ca^2+^ influx amplitude (Δ*F*/*F*_0 max_) at single AZs is reduced in IHCs of Ca_V_1.3^AG/AG^ mice. (**C**) Fractional activation curves of Ca^2+^ channels at individual AZs. Thick, dark lines show the averages, and lighter colors represent individual curves. (Ca and Cb) Box plots showing *k* (Ca) and *V*_half_ (Cb) of Ca^2+^ channels calculated from the Boltzmann fits in (C). (**D**) The spatial gradient of maximal Ca^2+^ influx is collapsed in Ca_V_1.3^AG/AG^ IHCs. n.s., not significant. (**E**) The spatial gradient of *V*_half_ is maintained in Ca_V_1.3^AG/AG^ IHCs. Polar plots in (D) and (E) show the positions of individual AZs in Ca_V_1.3^WT/WT^ (left) and Ca_V_1.3^AG/AG^ (right) IHCs. Pseudocolor scales represent maximal Ca^2+^ influx amplitude (D) and *V*_half_ (E). Box plots compare Δ*F*/*F*_0 max_ (D) and *V*_half_ (E) at the pillar and modiolar AZs in IHCs of Ca_V_1.3^WT/WT^ and Ca_V_1.3^AG/AG^ mice. Data were acquired from *N* = 8 (Ca_V_1.3^WT/WT^) and 6 (Ca_V_1.3^AG/AG^) mice. Box-whisker plots with individual data points overlaid show the median, 25th, and 75th percentiles (box) and 10th and 90th percentiles (whiskers). Statistical significances were determined using two-tailed Wilcoxon rank sum test for data in (Ba), (Ca), (Cb), (D), and (E). Significances are reported as *P < 0.05, **P < 0.01, and ***P < 0.001.

We then used spinning disk confocal Ca^2+^ imaging to analyze Ca^2+^ channel function at single IHC AZs (*17*, *18*). We identified single AZs by labeling their ribbons with a Ribeye/Ctbp2-binding fluorescently labeled peptide (*8*, *48*) and then recorded the change in fluorescence of the green Ca^2+^ indicator Fluo4-FF [dissociation constant (*K*_d_): 10 μM; Fig. 2A]. The Ca^2+^ indicator signal, under the strongly Ca^2+^-buffered condition (10 mM EGTA in the pipette), provides a good proxy of Ca^2+^ influx at the AZ (*8*, *49*). In keeping with the reduction of Ca_V_1.3 channel number of IHC AZs (fig. S3) but in contrast to the whole-cell Ca^2+^ current, we found a reduction of the maximal amplitude of the Ca^2+^ signals [Δ*F*/*F*_0 max_, approximating the maximal AZ Ca^2+^ influx (*8*)] of Ca_V_1.3^AG/AG^ AZs (Fig. 2, B and Ba). AZs of Ca_V_1.3^AG/AG^ IHCs showed a −15-mV shift of the voltage of half-maximal activation (*V*_half_; Fig. 2, C and Cb) and increased voltage sensitivity of Ca^2+^ influx activation (Fig. 2Ca), in agreement with the whole IHC recordings.

Next, we compared the properties of synapses of the modiolar and pillar IHC sides and found the modiolar-pillar gradient of maximal AZ Ca^2+^ influx (greater Δ*F*/*F*_0 max_ for modiolar synapses) typical for wild-type (WT) IHCs (*17*) to be collapsed in Ca_V_1.3^AG/AG^ IHCs (Fig. 2D). This finding was contrasted by a reduced, yet significant modiolar-pillar gradient of Ca_V_1.3 immunofluorescence intensity (fig. S4). This, together with the modiolar-pillar gradient of Ribeye/Ctbp2 immunofluorescence, indicates that Ca_V_1.3^AG/AG^ IHCs at least partially maintain larger ribbons with greater complement of Ca_V_1.3 for modiolar AZ. An opposing (modiolar-pillar) gradient is typically found for the voltage dependence of Ca^2+^ influx at the AZ with lower *V*_half_ at pillar synapses (*17*) that was also present in Ca_V_1.3^AG/AG^ IHCs (Fig. 2E) and further evident from correlations of *V*_half_ and synapse position along the pillar-modiolar axis (fig. S5).

**Fig. 3.**
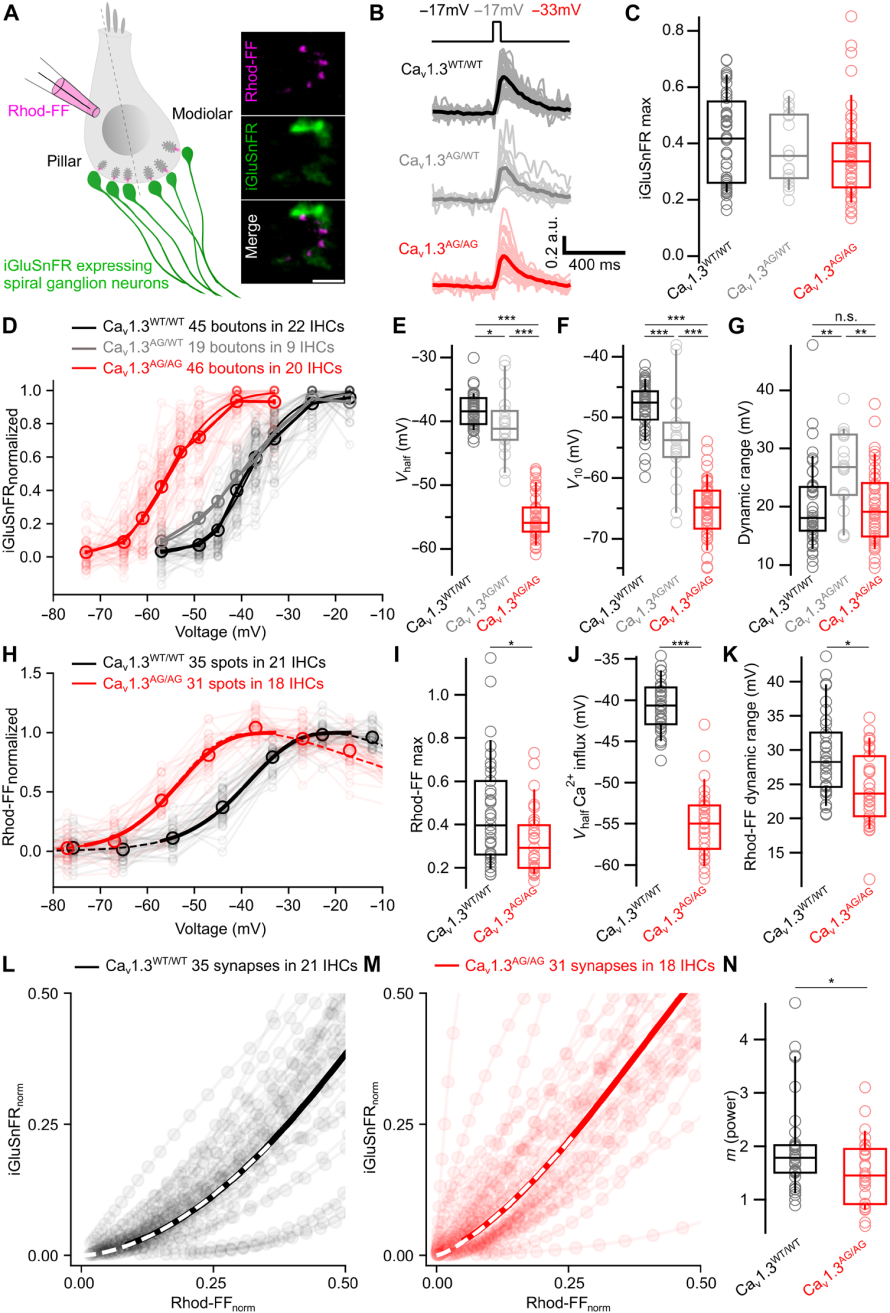
Activation of glutamate release at IHC synapses occurs at lower voltages in Ca_V_1.3^AG^ mice. (**A**) Average projection of Δ*F* images of Rhod-FF fluorescence from multiple planes of a representative Ca_V_1.3^WT/WT^ IHC (top). Average projection of Δ*F* images of iGluSnFR fluorescence from 50-ms depolarizations at a single IHC plane (middle). Scale bar, 5 μm. (**B**) Average Δ*F*/*F*_0_ traces of iGluSnFR fluorescence. Individual traces are in lighter colors. (**C**) Box plot showing Δ*F*/*F*_0 max_ of iGluSnFR signal. (**D**) Voltage dependence of normalized iGluSnFR signal. Data from individual boutons are shown for Ca_V_1.3^WT/WT^ and Ca_V_1.3^AG/AG^ animals. (**E** to **G**) Box plots showing *V*_half_ (E), *V*_10_ (F), and dynamic range (G) of glutamate release. (**H**) Voltage dependence of normalized Ca^2+^ influx at single AZs. Individual traces are shown with lighter colors. Dotted lines show the average modified Boltzmann function fit. Thick lines show fits matching the voltage dependency range of iGluSnFR recordings. (**I** to **K**) Box plots showing Δ*F*/*F*_0 max_ (I), *V*_half_ (J), and dynamic range (K) of Ca^2+^ influx. (**L** and **M**) Synaptic transfer function at single synapses in Ca_V_1.3^WT/WT^ (L) and Ca_V_1.3^AG/AG^ (M) IHCs. A thick, solid line shows the average. Dotted line represents the power function fitted to the first 25% of the glutamate release. (**N**) Box plot showing power (*m*) of Ca^2+^ influx–glutamate release. Data were acquired from *N* = 12 (Ca_V_1.3^WT/WT^), 5 (Ca_V_1.3^AG/WT^), and 9 (Ca_V_1.3^AG/AG^) mice. Box-whisker plots with individual data points overlaid show the median, 25th, and 75th percentiles (box) and 10th and 90th percentiles (whiskers). Statistical significances were determined using the Kruskal-Wallis test for (C), one-way ANOVA followed by Tukey’s post hoc test for (E) and (F), Kruskal-Wallis followed by Dunn’s test for (G), two-tailed Wilcoxon rank sum test for (I) and (N), and two-tailed *t* test for (J) and (K). Significances are reported as *P < 0.05, **P < 0.01, and ***P < 0.001.

To assess the consequences of the reduced number and hyperpolarized activation of Ca^2+^ channels for glutamate release at IHC AZs, we expressed the glutamate sensor iGluSnFR (*50*) in SGN terminals (Fig. 3A) (*18*). The iGluSnFR signal evoked by strong depolarizations (50 ms to −17 mV for Ca_V_1.3^WT/WT^/Ca_V_1.3^AG/WT^ and −33 mV for Ca_V_1.3^AG/AG^) of Ca_V_1.3^AG/AG^ synapses (iGluSnFR_max_) tended to be lower than that of Ca_V_1.3^WT/WT^ synapses without reaching statistical significance (Ca_V_1.3^AG/WT^ fell in between) (Fig. 3, B and C). In contrast to this, but consistent with results obtained with Fluo4-FF dye, the maximal presynaptic Ca^2+^ influx of the same AZs reported with the red Ca^2+^ indicator Rhod-FF (*K*_d_: 320 μM) was significantly reduced for Ca_V_1.3^AG/AG^ IHCs (Fig. 3I). The operating range of glutamate release was shifted to lower voltages in Ca_V_1.3^AG/AG^ synapses (−18-mV shift of *V*_half_; Fig. 3E). We detected significant iGluSnFR signals in Ca_V_1.3^AG/AG^ mice with depolarizations as weak as −65 mV (*V*_10%_; Fig. 3, D and F) compared to −53 mV for Ca_V_1.3^AG/WT^ and −47 mV for Ca_V_1.3^WT/WT^ mice.

The activation curves of Ca_V_1.3^AG/WT^ and Ca_V_1.3^WT/WT^ synapses differed only for depolarizations to less than −40 mV, which might result from differences of the Ca_V_1.3^AG/WT^ synapses in expressing WT and AG channels. This could also explain the greater dynamic range of the transfer function found with Ca_V_1.3^AG/WT^ synapses compared to the other genotypes (Fig. 3G). By summing fractions of whole-cell Ca^2+^ channel activation curves (Fig. 1C) from Ca_V_1.3^WT/WT^ and Ca_V_1.3^AG/AG^ IHCs, we found that combining 83% WT channels and only 17% AG channels best explained voltage-dependent activation of Ca^2+^ currents in Ca_V_1.3^AG/WT^ IHCs. We found the voltage dependence of the iGluSnFR signal to closely follow that of Ca^2+^ channel activation for Ca_V_1.3^WT/WT^ and Ca_V_1.3^AG/AG^ synapses (Fig. 3, D to H, J, and K).

Next, we related the iGluSnFR signals obtained for different levels of IHC depolarization to the corresponding Ca^2+^ signals to characterize the apparent Ca^2+^ dependence of glutamate release. We note that this protocol grades Ca^2+^ influx primarily via changing the open probability and, to a lesser extent, via the single channel current. The lower power (*m =* 1.5) compared to that of the intrinsic Ca^2+^ dependence of exocytosis (*m* = 2.5) at synapses of WT mice (*18*) is thought to reflect a tight Ca^2+^ nanodomain–like control of SV release by one or few Ca^2+^ channels with an effective coupling distance of ~15 nm (*51*, *52*). Ca^2+^ nanodomain–like control of SV release was maintained with even lower power (Ca_V_1.3^AG/AG^: *m =* 1.51 ± 0.11 versus Ca_V_1.3^WT/WT^: *m =* 1.95 ± 0.15; *P* = 0.042), hence tighter coupling of Ca^2+^ influx and release at Ca_V_1.3^AG/AG^ AZs (Fig. 3, L to N).

### Increased spontaneous firing in SGNs of Ca_V_1.3^AG^ animals

**Fig. 4.**
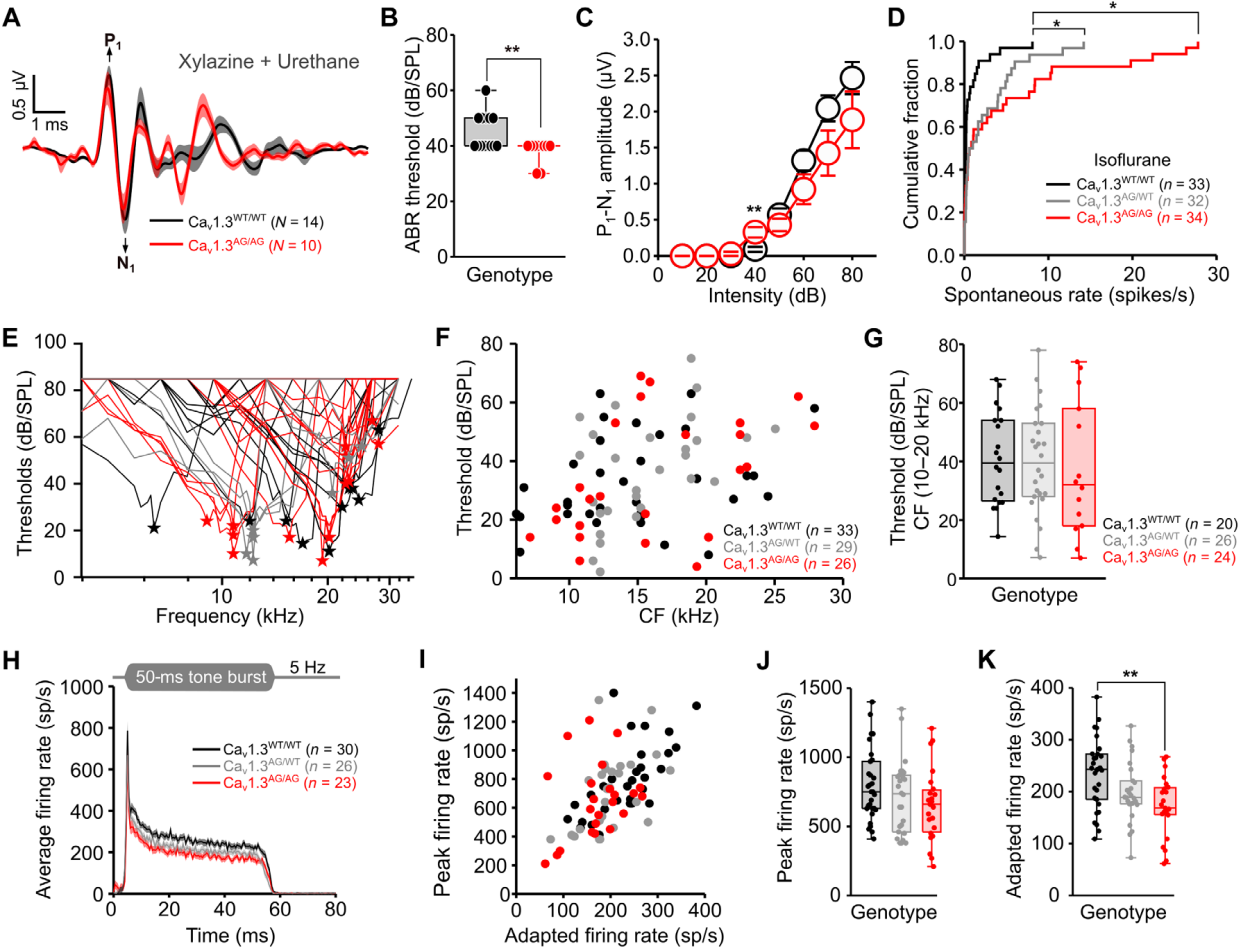
Increased spontaneous rates in SGNs of Ca_V_1.3^AG/WT^ and Ca_V_1.3^AG/AG^ mice. (**A**) Average ABR waveforms in response to 80-dB clicks recorded in mice under urethane/xylazine anesthesia. Shaded areas show ± SEM. (**B**) ABR thresholds in response to click stimuli are lower in Ca_V_1.3^AG/AG^ mice compared to Ca_V_1.3^WT/WT^ littermates. SPL, sound pressure level. (**C**) ABR P_1_-N_1_ amplitude was significantly bigger in Ca_V_1.3^AG/AG^ at near-threshold stimulation [40 dB (peak equivalent)] but similar to Ca_V_1.3^WT/WT^ across other sound pressure levels. (**D**) SRs of SGNs recorded from mice under isoflurane anesthesia show a relative increase in SRs in Ca_V_1.3^AG/WT^ and Ca_V_1.3^AG/AG^ mice compared to Ca_V_1.3^WT/WT^. (**E**) Frequency tuning curves of all recorded putative SGNs with the characteristic frequency (CF)/best threshold marked by stars. (**F** and **G**) Thresholds at CF between 10 and 20 kHz (G) are comparable in Ca_V_1.3^WT/WT^, Ca_V_1.3^AG/WT^, and Ca_V_1.3^AG/AG^ mice. (**H**) Average peristimulus time histogram (PSTH) in response to 50-ms stimulation at the CF, 30 dB above the threshold level, and stimulation rate of 5 Hz. Shaded areas show ± SEM. (**I** to **K**) Onset firing rates (calculated from PSTH as the bin with the highest rate at the sound onset) are not changed (J), but the adapted firing rates (averaged firing rates at 35 to 40 ms after the sound onset) are decreased in SGNs of Ca_V_1.3^AG/AG^ mice (K). Single-unit recordings were obtained from *N* = 6 (Ca_V_1.3^WT/WT^), 3 (Ca_V_1.3^AG/WT^), and 6 (Ca_V_1.3^AG/AG^) mice. Box-whisker plots with individual data points overlaid show the median, 25th, and 75th percentiles (box) and the range (whiskers). Statistical significances were determined using two-tailed Wilcoxon rank sum test for (B), two-tailed Wilcoxon rank sum test for each sound level for (C), Kruskal-Wallis test followed by Tukey-Kramer multiple comparison test for (D) and (K), and Kruskal-Wallis test for (G) and (J). Significances are reported as *P < 0.05 and **P < 0.01.

Next, we addressed the impact of shifting the synaptic transfer function of Ca_V_1.3^AG/AG^ synapses to lower voltages on spontaneous and sound-evoked firing of SGNs in vivo*.* We first recorded auditory brainstem responses (ABRs) in response to acoustic clicks (Fig. 4A). The first wave (wave I) reports the firing of the SGN population, and its amplitude reflects both the number of activated SGNs and their firing synchrony. ABR threshold was significantly lower in Ca_V_1.3^AG/AG^ mice than in Ca_V_1.3^WT/WT^ mice, which is consistent with the lower activation threshold of Ca_V_1.3^AG^ channels (Fig. 4B). Wave I amplitude was mildly but significantly increased at near-threshold 40-dB acoustic click stimulation yet not systematically changed across the higher sound pressure levels in Ca_V_1.3^AG/AG^ mice (Fig. 4C). This suggests increased firing rates of SGNs for weak stimuli, while steady-state inactivation of the channels might limit synaptic transmission at higher sound pressure levels.

Ca_V_1.3^AG/AG^ mice did not very well tolerate the urethane/xylazine anesthesia typically used for the demanding stereotactic recordings from single SGNs (*13*, *53*). Hence, we turned to isoflurane anesthesia but note that isoflurane inhibits voltage-gated Ca^2+^ channels (*54*) and we cannot exclude that the higher open probability renders Ca_V_1.3^AG^ channels more susceptible to isoflurane. This might explain why ABR thresholds and wave I amplitudes of both genotypes were not significantly different from each other under isoflurane (fig. S7, A to C). Moreover, SRs were strongly reduced in WT SGNs recorded in isoflurane compared to urethane/xylazine (fig. S7D).

Despite all this, SR was increased in Ca_V_1.3^AG/AG^ mice and even in Ca_V_1.3^AG/WT^ mice (Fig. 4D and fig. S6). Sound-evoked SGN firing was largely intact: Frequency tuning and thresholds (Fig. 4, E to G, and fig. S7L) and peak firing rates (Fig. 4, H to J) were not significantly altered in Ca_V_1.3^AG/AG^ or Ca_V_1.3^AG/WT^ mice. Adapted spike rates in Ca_V_1.3^AG/AG^ SGNs were reduced, likely reflecting enhanced inactivation of Ca_V_1.3^AG^ channels (Fig. 4, H, I, and K), which was further corroborated by probing 1.5-s-long tone bursts (fig. S7, E to H). The dynamic range of the rate code was comparable across the genotype with a nonsignificant trend toward larger ranges for Ca_V_1.3^AG/WT^ SGNs, which would be as expected for AZs mixing Ca_V_1.3^AG^ and Ca_V_1.3^WT^ channels (fig. S7, I to K). In summary, at least when analyzed in isoflurane, it appears that the cochlea manages to maintain sound encoding at control conditions, despite a massive change in Ca_V_1.3 gating that manifests itself at the level of spontaneous synaptic transmission and SGN firing around the sound threshold.

### Loss of synaptic ribbons in IHCs of Ca_V_1.3^AG/AG^ mice at ambient sound levels

On the basis of our findings in Ca_V_1.3^AG/WT^ mice, we postulate higher acoustic sensitivity or normal hearing in patients affected by the Ca_V_1.3^AG^ mutation. Inspired by the gain of IHC AZ function and increased spontaneous SGN firing in Ca_V_1.3^AG/WT^ mice, we addressed the clinically relevant question if patients might face a higher risk of noise-induced synaptopathy (*42*–*44*). This so-called hidden hearing loss is not detectable by common clinical hearing tests of acoustic sensitivity, because sound encoding near threshold involves high SR fibers that are better maintained upon noise exposure (*43*). We turned to electron and immunofluorescence microscopy (EM and confocal microscopy) to study the number and morphology of IHC synapses across the cochlear turns in Ca_V_1.3^AG/AG^ mice around the onset of hearing and in 1- to 2-month-old adults, when rearing in quiet (see Materials and Methods) and standard acoustic environment of the animal facility.

**Fig. 5.**
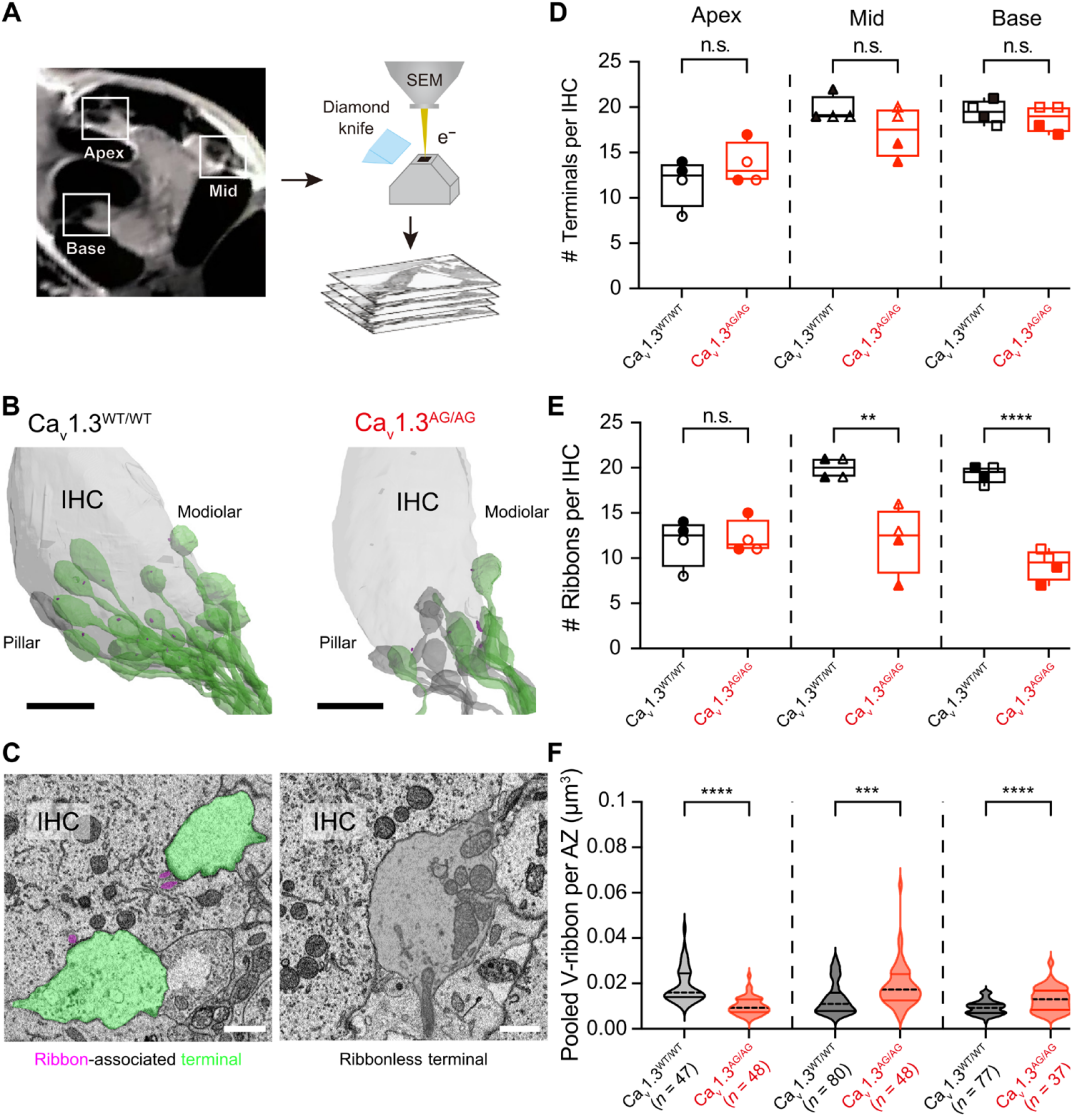
Loss of synaptic ribbons at a subset of IHC AZs in Ca_V_1.3^AG/AG^ mice. (**A**) Schematic illustration of SBEM imaging at apical, middle, and basal segments of the mouse cochlea. (**B**) Three-dimensional (3D) rendering of reconstructed Ca_V_1.3^WT/WT^ (left) and Ca_V_1.3^AG/AG^ (right) IHCs contacted by SGN terminals with (green) and without (gray, “ribbonless”) associated ribbons (magenta). Scale bars, 5 μm. (**C**) Representative electron micrographs of ribbon-associated (green; left) and ribbonless (gray; right) SGN terminals. Scale bars, 1 μm. (**D**) Average numbers of SGN terminals contacting an IHC are comparable between Ca_V_1.3^AG/AG^ and Ca_V_1.3^WT/WT^ mice. (**E**) Average ribbon counts are significantly smaller in Ca_V_1.3^AG/AG^ IHCs compared to Ca_V_1.3^WT/WT^ at mid- and basal cochlear segments but not in the apex. (**F**) Mean volumes of ribbons are larger in Ca_V_1.3^AG/AG^ IHCs compared to Ca_V_1.3^WT/WT^ IHCs at mid- and basal cochlear segments, whereas apical Ca_V_1.3^AG/AG^ IHCs appear to have exclusively small ribbons. Each tonotopic location of each genotype represents data from *N* = 2 mice. Box-whisker plots with individual data points overlaid show median, 25th, and 75th percentiles (box) and the range (whiskers). Statistical significances were determined using two-tailed *t* test for (D) to (F). Significances are reported as **P < 0.01, ***P < 0.001, and ****P < 0.0001.

When reconstructing IHCs of various cochlear turns of normally reared Ca_V_1.3^AG/AG^ and Ca_V_1.3^WT/WT^ mice by serial block-face scanning EM (SBEM; Fig. 5, A and B), we found a comparable number of postsynaptic SGN terminals contacting the IHC between two groups (Fig. 5D). However, in the mid- and basocochlear regions, only half of the SGN terminals contacting Ca_V_1.3^AG/AG^ IHCs were associated with synaptic ribbons (Fig. 5, C and E). In agreement with the immunofluorescence data (fig. S3B), the ribbons of apical IHCs were smaller, whereas the remaining ribbons of mid- and basocochlear IHCs were significantly larger (Fig. 5F). We further assessed the morphology of the ribbons by transmission EM and found a higher abundance of ribbons with a hollow core (fig. S8).

In Ca_V_1.3^AG/AG^ animals, we found 73% of the total ribbonless terminals in the midcochlear region and 76% in the basocochlear region to contact the pillar side of IHCs, likely reflecting higher activity of the pillar AZs compared to the modiolar ones due to more hyperpolarized Ca^2+^ influx. Immunohistochemistry confirmed the observations of fewer synaptic ribbons for 2- and 9-month-old mice (fig. S9, B, Ba, C, and Ca). It also revealed that Ca_V_1.3^AG/AG^ IHCs start out with a normal complement of ribbons at the onset of hearing (fig. S9, A and Aa) and maintain them better when quiet reared in particular in the basal cochlea (fig. S9, D and Da). To probe for potential loss of ribbons in the Ca_V_1.3^AG/WT^ mouse model of human patients, we performed immunohistochemistry in whole mounts of the organ of Corti at the age of 2 months. We did not observe a change in the number of ribbons in IHCs of apical, middle, and basal cochlear turns (fig. S10).

Next, we quantified the mitochondrial content of SGNs in our SBEM reconstructions to check for potential changes due to increased presynaptic or SGN activity in Ca_V_1.3^AG/AG^ animals (fig. S11A). We observed an increase in mitochondrial volume and density in both the terminals and peripheral neurites of SGNs in the cochlear mid-turn of Ca_V_1.3^AG/AG^ mice (fig. S11, B to D). Ribbon-associated terminals showed larger volumes and more mitochondria compared to the ribbonless terminals (fig. S11, E to H).

## DISCUSSION

Here, we analyzed a mouse line harboring the human p.A749G point mutation in Ca_V_1.3 channels. We observed a robust hyperpolarized shift of the voltage of activation for Ca^2+^ channels and glutamate release at the AZs of IHCs. IHC AZs of Ca_V_1.3^AG/AG^ mice showed about 40% of the maximal glutamate release at −58 mV, around the resting membrane potential of the cells (*16*). This compares to about 10% at IHC AZs of Ca_V_1.3^AG/WT^ mice, which we estimated to have 83% WT and 17% mutant channels, and no detectable release at WT IHC AZ. Concomitantly, the distribution of the SRs in SGNs was shifted to higher rates in Ca_V_1.3^AG/WT^ and more markedly in Ca_V_1.3^AG/AG^ mice. Together, this indicates that the voltage dependence of Ca^2+^ channel activation in IHCs governs SRs in SGNs. Furthermore, in urethane/xylazine anesthesia, Ca_V_1.3^AG/AG^ mice displayed mildly reduced ABR thresholds and increased ABR wave I amplitudes at low sound pressure levels, suggesting that Ca_V_1.3 activation at lower voltages also enhances encoding of soft sounds.

The impact of the voltage dependence of Ca_V_1.3 activation on spontaneous and sound-evoked SGN firing could have been underestimated in the present study for several reasons. First, as shown in heterologous expression data, along with the activation, the inactivation of Ca_V_1.3^AG^ channels is shifted toward hyperpolarized potentials (*7*). This could potentially limit the number of channels available for activation around the resting membrane potential of the IHCs, directly affecting SR and sound encoding in SGNs (*39*, *40*). It would be interesting for future studies to address Ca_V_1.3 mutations that change activation without majorly affecting the inactivation (*37*, *55*). Second, the observed reduction in the Ca_V_1.3 complement of IHC AZs in Ca_V_1.3^AG/AG^ further limited the number of Ca^2+^ channels available for spontaneous and sound-evoked synaptic transmission to SGNs.

Last, using isoflurane anesthesia for the invasive recordings from single SGNs likely limited the number of Ca^2+^ channels available for spontaneous and sound-evoked synaptic transmission. Isoflurane has been reported to inhibit the activity of the L-type Ca^2+^ channels (*54*). More recently, it was shown that the direct application of isoflurane to IHCs reversibly reduces Ca^2+^ current amplitude and potentially affects exocytosis (*56*). While the mechanisms of the isoflurane block are not known, pharmacological agents such as dihydropyridines or nondihydropyridine drugs are highly state dependent and preferentially bind to the active or inactive states of the channels (*57*). Therefore, Ca_V_1.3^A749G^ channels are likely more sensitive to isoflurane inhibition. The acoustic thresholds estimated under urethane/xylazine anesthesia were significantly lower in Ca_V_1.3^AG/AG^ mutants compared to the controls, while under the isoflurane anesthesia, no difference was observed. Hence, further probing the impact of Ca_V_1.3^AG^ on sound encoding will benefit from identifying anesthesia tolerated by Ca_V_1.3^AG^ mice that does not affect Ca_V_ channels.

**Fig. 6.**
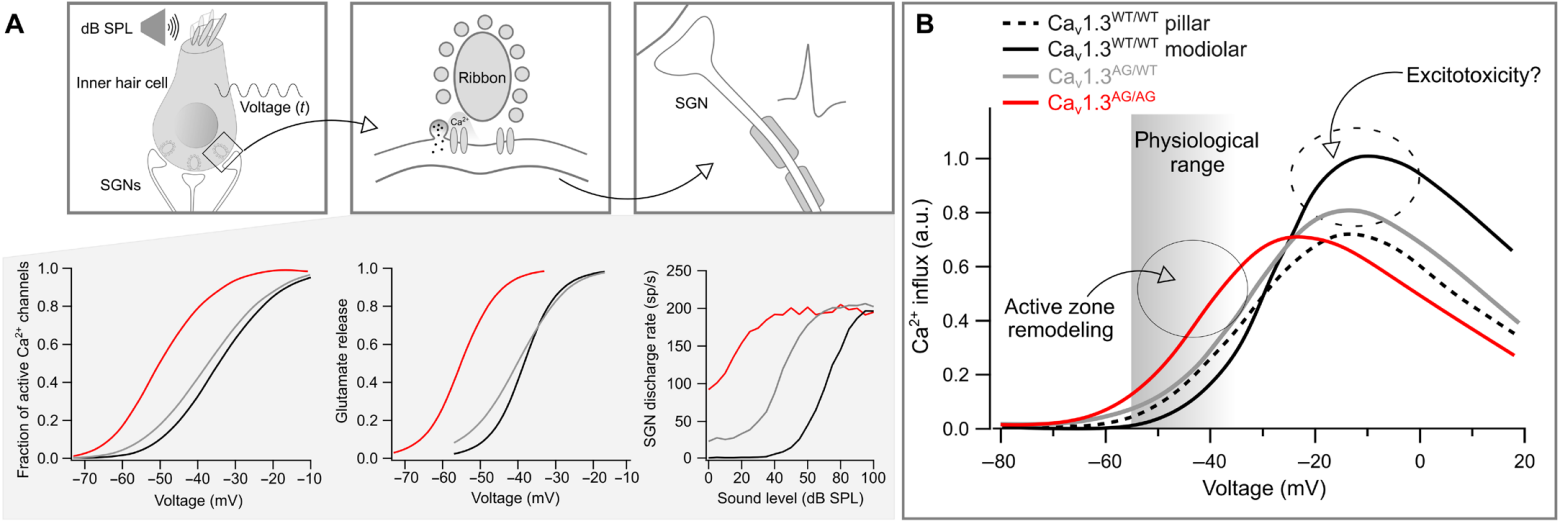
Ca_V_1.3 gating contributes to SGN firing diversity and synaptic vulnerability. (**A**) SGN spontaneous rates and thresholds vary according to the voltage dependence of presynaptic Ca^2+^ channel activation and glutamate release in a gene dose–dependent manner. SGN rate-level functions were modeled according to Meddis *et al.* (*58*) (see Supplementary Text, fig. S12, and table S1). Top panels summarize the path from sound to SGN code. (**B**) Hyperpolarized activation of Ca_V_1.3 channels in Ca_V_1.3^AG/AG^ mice leads to Ca^2+^ influx at IHC AZs, exceeding that for both pillar and modiolar AZs in WT IHCs at physiological voltages. This results in a homeostatic reduction of Ca^2+^ channels and a general AZ remodeling but does not reach excitotoxic levels of Ca^2+^ influx and glutamate release at the ambient sound levels of the animal facility. Excitotoxic damage is likely to occur at large receptor potentials during intense sound stimulation and may preferentially affect modiolar synapses that harbor a greater Ca^2+^ channel complement in WT IHCs at nonphysiological stimulations, such as moderate noise exposure (shown with the dashed ellipsoid).

In conclusion, the increased SGN SR—evident even under isoflurane—and reduced ABR threshold (urethane/xylazine) demonstrate the impact of presynaptic Ca^2+^ channel gating in IHCs on spontaneous and sound-evoked SGN firing (Fig. 6A). The graded hyperpolarized shift of IHC synapse activation and ensuing increase in spontaneous SGN firing demonstrated here for Ca_V_1.3^AG^ in a gene dose–dependent manner lend support for the hypothesis that the physiologically observed differences of AZs in Ca_V_1.3 activation contribute to SGN firing diversity. We used computational modeling based on an established framework (*58*, *59*) to further test this hypothesis (Fig. 6A, fig. S12, and table S1). The model confirmed the hypothesis for both spontaneous and sound-evoked SGN firing. Obviously, auditory threshold is co-determined by the passive and active micromechanics of the ear (*60*), the endocochlear potential (*15*), and the sensitivity of mechanotransduction in hair cells (*61*), which are approximated in this model. Hence, there is a lower limit for Ca_V_1.3 activation to affect the auditory threshold.

Ca_V_1.3^AG/AG^ IHCs retained the pillar-modiolar AZ gradient of voltage operating range despite the massive hyperpolarized shift. Hence, while Ca_V_1.3^AG^ IHCs homeostatically and likely cell-autonomously down-regulate the abundance of Ca_V_1.3 at AZs, the position-dependent regulation of the voltage dependence of Ca_V_1.3 activation does not seem to target absolute values and might involve cell-nonautonomous signaling.

We found that increased activity of Ca^2+^ channels in Ca_V_1.3^AG/AG^ IHCs leads to partial loss of synaptic ribbons even at the ambient noise levels of standard animal husbandry, while potential excitotoxic damage to SGN terminals was not evident. We cannot rule that excitotoxic damage to SGNs occurred but was repaired by axonal regrowth and the formation of new synapses. However, we consider the partial loss of ribbons more likely to represent a selective presynaptic remodeling that might involve Ca^2+^-dependent mitochondrial signaling to ribbons (Fig. 6B) (*62*, *63*) and goes hand in hand with a homeostatic reduction of Ca_V_1.3 channels (figs. S2 and S3). SGNs showed an increase in the total mitochondrial volumes in their postsynaptic terminals and peripheral neurites in Ca_V_1.3^AG/AG^ that we consider to reflect increased synaptic transmission and/or SGN activity (*32*). An increase in mitochondrial content of SGN terminals was also shown to be caused by noise exposure (*44*). While we did not find a significant ribbon loss in the Ca_V_1.3^AG/WT^ mouse model of human patients, we caution that their ears could be more susceptible to noise-induced hearing loss (Fig. 6B). Future work will need to test this hypothesis with acute and chronic exposure of the mouse model to different noise levels. Moreover, testing auditory function in patients with gain-of-function mutations in *CACNA1D*, ideally in a longitudinal manner, is an important objective for future studies. In summary, our findings support the hypothesis that the voltage dependence of Ca_V_ gating at a given IHC-SGN synapse governs spontaneous and sound-evoked SGN firing and indicate that heterogeneity of Ca_V_ gating among the synapses contributes to SGN diversity.

## MATERIALS AND METHODS

### Animals

Ca_V_1.3^AG^ mice have been previously described (*36*). We used homozygous (Ca_V_1.3^AG/AG^) and heterozygous (Ca_V_1.3^AG/WT^) mutants and WT (Ca_V_1.3^WT/WT^) control mice of either sex for the experiments. Mice were maintained on the C57B6/N background. For certain patch-clamp experiments (capacitance measurements, tail current recordings, and patch clamp combined with Fluo4-FF imaging), C57B6J animals were used along with the littermate controls. Ages of the mice varied from 13 days to 9 months depending on the experiment. Animals were housed and raised either under standard husbandry conditions in individually ventilated cages or in open cages placed in an isolated quiet environment with low ambient noise levels, particularly lacking the sound of the ventilation system. All the experiments were approved by the local Animal Welfare Committee of the University Medical Center Göttingen and the Max Planck Institute for Multidisciplinary Sciences, as well as the Animal Welfare Office of the state of Lower Saxony, Germany (LAVES, AZ: 19/3134 and 19/3133).

### Patch clamp

For ex vivo physiology, we dissected three-fourths of the apical turn of the organ of Corti from 2- to 4-week-old mice. For the experiments, where the ruptured patch clamp was combined with Ca^2+^ imaging using Fluo4-FF dye, IHCs were accessed from the modiolar side to preserve the general morphology of the cells for later assignment of modiolar and pillar synapses. For the remaining experiments, IHCs were accessed from the pillar side of the organ. Patch pipettes were made using P-97 Flaming/Brown micropipette puller (Sutter Instruments) and borosilicate glass filaments [GB150-8P and GB150F-8P (Science Products), for perforated and ruptured patch-clamp configurations, respectively]. For perforated patch-clamp and capacitance recordings, pipette solution contained 130 mM Cs-gluconate, 10 mM Hepes, 10 mM tetraethylammonium (TEA)–Cl, 10 mM 4-aminopyridine (4AP), 1 mM MgCl_2_, amphotericin B (300 μg/ml), pH 7.3, 290 mosmol. For ruptured patch clamp combined with imaging, pipette solution contained 111 mM Cs-glutamate, 1 mM MgCl_2_, 1 mM CaCl_2_, 10 mM EGTA, 13 mM TEA-Cl, 20 mM Hepes, 4 mM Mg–adenosine 5′-triphosphate (ATP), 0.3 mM Na–guanosine 5′-triphosphate (GTP), 1 mM l-glutathione, pH 7.3, 290 mosmol. In addition, Ca^2+^ indicator Fluo4-FF (0.8 mM; Life Technologies) and 5-Carboxytetramethylrhodamin (TAMRA)-conjugated Ribeye/Ctbp2-binding peptide (10 mM; Biosyntan) or Rhod-FF (0.8 mM; Biomol) were added to the intracellular solution for live imaging. For ruptured patch clamp to record the variance of the tail currents, the pipette solution contained 130 mM Cs-gluconate, 1 mM MgCl_2_, 10 mM Hepes, 10 mM TEA, 0.8 mM EGTA, 0.4 mM 1,2-bis(2-aminophenoxy)ethane-*N*,*N*,*N′*,*N′*-tetraacetic acid (BAPTA), 10 mM 4AP, 2 mM Mg-ATP, 0.3 mM Na_2_-GTP, d-glucose (2 mg/ml), pH 7.2, 300 mosmol. Perforated patch clamp was performed in extracellular solution containing 107 mM NaCl, 2.8 mM KCl, 1 mM MgCl_2_, 10 mM Hepes, 2 mM CaCl_2_, 35 mM TEA, 5 mM 4AP, 1 mM CsCl, d-glucose (2 mg/ml), pH 7.2, 300 mosmol. Ruptured patch clamp combined with imaging was performed in extracellular solution containing 2.8 mM KCl, 105 mM NaCl, 10 mM Hepes, 1 mM CsCl, 1 mM MgCl_2_, 5 mM CaCl_2_, 35 mM TEA-Cl, d-glucose (2 mg/ml), pH 7.2, 300 mosmol. Ruptured patch clamp for recording the variance of the tail Ca^2+^ currents was performed in extracellular solution containing 90 mM NaCl, 2.8 mM KCl, 1 mM MgCl_2_, 10 mM Hepes, 10 mM CaCl_2_, 35 mM TEA, 1 mM CsCl, 5 μM Bay K8644, glucose (2 mg/ml), 300 mosmol. Data acquisition was done using an EPC-10 amplifier (HEKA Electronics) controlled by the PATCHMASTER software (HEKA Electronics). The holding potential of IHCs was set to −97 mV. All recordings were performed at room temperature (RT; 20° to 25°C).

#### Perforated patch clamp

Pipettes were coated with SYLGARD to minimize the capacitive noise. Capacitance measurements from IHCs were performed using the Lindau-Neher technique (*64*) as described previously (*65*). Current-voltage (*I*-*V*) relationships were recorded by applying 10-ms step depolarizations ranging from −97 to 63 mV with 5-mV increments. Recordings were leak corrected using the p/n protocol. All voltages were corrected offline for the liquid junction potential (17 mV). Recordings where series resistance (*R*_s_) exceeded 30 megohms, leak currents exceeded −50 pA at the holding potential, and Ca^2+^ current rundown was more than 25% were discarded from the analysis. To analyze capacitance changes, traces were averaged 400 ms before and after depolarization (skipping 60 ms of the initial segment after depolarization).

#### Ruptured patch clamp

Voltage ramp depolarizations ranging from −97 to 53 mV or −97 to 63 mV during 150 ms were applied to the cells. Leak correction was done using the p/n protocol, and the liquid junction potential of 17 mV was corrected offline. Recordings were discarded from the analysis if *R*_s_ exceeded 14 megohms during the first 3 min after breaking into the cell, leak current exceeded −50 pA at holding potential, and Ca^2+^ current rundown was more than 25%. For glutamate imaging, 50-ms step depolarizations to various voltages were applied to the cells in a pseudorandom order. The recording and analysis of the variance of Ca^2+^ tail current were performed as described before (*66*).

### Functional imaging

Functional imaging was performed using a spinning disk confocal unit (CSU22, Yokogawa) mounted on an upright microscope (Axio Examiner, Zeiss). The spinning disk was set to 2000 rpm. We used 63×, 1.0 numerical aperture (NA) objective (W Plan-Apochromat, Zeiss), and images were acquired with a scientific complementary metal-oxide semiconductor camera (Andor Neo), with a pixel size of 103 nm. The setup was further equipped with 491-nm (Calypso, Cobolt AB) and 561-nm (Jive, Cobolt AB) lasers. A Piezo positioner (Piezosystem) was used to acquire images at different *z* planes.

#### Fluo4-FF and TAMRA imaging

IHCs were loaded with Fluo4-FF Ca^2+^ dye and TAMRA-conjugated Ribeye/Ctbp2-binding dimeric peptide via the patch pipette. First, the cells were scanned from bottom to top by imaging TAMRA fluorescence with a 561-nm laser with a 0.5-s exposure time and a 0.5-μm step size. This allowed us to obtain cell morphology and visualize synaptic ribbons. Next, we recorded Fluo4-FF fluorescence increase at individual synapses by imaging ribbon-containing planes with a 491-nm laser at 100 Hz while applying voltage ramp depolarizations to the cell. Two voltage ramps were applied at each plane, one being 5-ms shifted relative to the other.

#### iGluSnFR and Rhod-FF imaging

Genetically encoded glutamate sensor iGluSnFR was targeted to SGNs by postnatal round window injections in p5 to p7 mice using the adeno-associated virus serotype 9 (AAV9), where iGluSnFR is expressed under the human synapsin promoter (pAAV9.*hSyn*.iGluSnFR.WPRE.SV40, Addgene or produced in our laboratory), as previously described (*18*). Because of the high density of synapses at the basal planes of IHCs, it is difficult to distinguish single postsynaptic boutons from one another. For that reason, we chose to image planes closer to the cell nucleus. Once the plane containing iGluSnFR-expressing postsynaptic boutons was located (central plane), we performed Ca^2+^ imaging at the central plane as well as two planes above and two planes below the central plane (step size = 0.5 μm). Rhod-FF fluorescence increase was evoked by two identical voltage ramp depolarizations at each of the five planes and was imaged using a 591-nm laser at 100 Hz. Afterward, iGluSnFR fluorescence was imaged at the central plane with a 491-nm laser at 50 Hz while stimulating the cell with 50-ms depolarizations of the following voltages in pseudorandom order: −57 mV, −49 mV, −45 mV, −41 mV, −37 mV, −33 mV, −25 mV, −17 mV for Ca_V_1.3^WT/WT^ and Ca_V_1.3^AG/WT^ and −73 mV, −65 mV, −61 mV, −57 mV, −53 mV, −49 mV, −41 mV, −33 mV for Ca_V_1.3^AG/AG^ mice.

### Immunohistochemistry and imaging

Cochleae from animals aged 13 days to 9 months were fixed with 4% formaldehyde on ice either for 45 min to 1 hour or for 10 min whenever Ca_V_1.3 channels were immunolabeled for the purpose of performing STED imaging. Cochleae from 1-month-old mice were fixed in glyoxal solution for 30 min on ice, followed by 30 min at RT whenever Ca_V_1.3 channels were stained for analyzing modiolar-pillar gradient of Ca_V_1.3 cluster sizes. Glyoxal fixation has been described before (*67*). Cochleae, which were used to count the presynaptic ribbons along the tonotopic axis, were further decalcified in EDTA (10%; pH 8) before dissecting apical, middle, and basal turns of the organ of Corti.

The following primary antibodies were used: rabbit anti-Homer1 (1:500; 160 002, Synaptic Systems), mouse anti-Ctbp2 (1:200; 612044, BD Biosciences), rabbit anti-Ca_V_1.3 (1:100; ACC-005, Alomone Labs), mouse anti-bassoon (1:300; ab82958, Abcam), guinea pig anti-RibeyeA (1:500; 192 104, Synaptic Systems), guinea pig anti-Vglut3 (1:500; 135 204, Synaptic Systems), and chicken anti-calretinin (1:200; 214 106, Synaptic Systems).

The following secondary antibodies were used: Alexa Fluor 488–conjugated anti–guinea pig (1:200; A11073, Thermo Fisher Scientific), Alexa Fluor 488–conjugated anti-rabbit (1:200; A11008, Thermo Fisher Scientific), Alexa Fluor 488–conjugated anti-mouse (1:200; A11001, Thermo Fisher Scientific), Alexa Fluor 568–conjugated anti-chicken (1:200; ab175711, Abcam), Alexa Fluor 568–conjugated anti–guinea pig (1:200; A11075, Thermo Fisher Scientific), Alexa Fluor 647–conjugated anti-rabbit (1:200; ab150079, Abcam), Alexa Fluor 647–conjugated anti-rabbit (1:200; A21244, Thermo Fisher Scientific), STAR 580–conjugated anti-mouse (1:200; ST635P-1001-500UG, Abberior), and STAR 635–conjugated anti-rabbit (1:200; ST635P-1002-500UG, Abberior).

Images were acquired using a 100× 1.4 NA oil immersion objective and Abberior Instruments Expert Line STED microscope equipped with 488-, 561-, and 633-nm lasers and a 775-nm STED laser. Z-stacks were acquired in confocal mode, while 2D imaging of the synapses was done in a 2D STED mode.

### In vivo recordings

#### Surgical procedure

Mice were anesthetized by intraperitoneal injection of xylazine (5 mg/kg) and urethane (1.32 g/kg) or by inhalation of isoflurane via a face mask (5 vol % in O_2_ for induction and 0.6 to 1.5 vol % in O_2_ for maintenance). Analgesia was achieved using buprenorphine (0.1 mg/kg; repeated every 4 hours) and, in the case of isoflurane anesthesia, additional carprofen (5 mg/kg; administered only once at the beginning of the experiment) administered subcutaneously. The animals were maintained at 37°C throughout the experiment using a custom-made heating pad and placed on a vibration isolation table in a soundproof chamber (IAC GmbH, Niederkrüchten, Germany). The depth of the anesthesia was regularly monitored by the absence of hindlimb withdrawal reflexes, and additional anesthetic doses were administered as needed. For the juxtacellular recordings from the auditory nerve, a tracheostomy was performed using a silicon tube to ensure breathing throughout the experiment. In the case of the isoflurane anesthesia, the face mask delivering the anesthetic was held in close proximity to the face and tracheal opening of the animals throughout this procedure. The pinnae were removed, after which the animals were rapidly positioned in a custom-designed stereotactic head holder, and a three-dimensional (3D)–printed adaptor was attached to the face mask to efficiently deliver the isoflurane directly to the tracheostomy tube until the end of the experiment. Next, the scalp was reflected, and part of the left occipital bone was removed. This procedure then allowed for a partial aspiration of the cerebellum to expose the anterior semicircular canal as a landmark for electrode positioning.

#### Auditory brainstem responses

The stimulus generation, presentation, data acquisition, and offline analysis were performed using the NI System (National Instruments, Austin, TX, USA) and the custom-written MATLAB software (MathWorks Inc.). The ABRs were recorded by needle electrodes placed subcutaneously near the pinna, on the vertex, and on the back near the hindlimbs.

The difference potential between vertex and mastoid subdermal needles was amplified 10,000 times using a custom-designed amplifier, sampled at a rate of 50 kHz for 20 ms, filtered (300 to 3000 Hz), and averaged across 500 presentations. Thresholds were determined by visual inspection as the minimum sound intensity that caused a reproducible response waveform in the recorded traces. The first ABR wave (P_1_-N_1_) was detected manually with a custom-written MATLAB script in which the wave was detected for each trace by the user.

#### Juxtacellular recordings from single putative SGNs

The procedure of juxtacellular recordings from SGNs has been described previously (*53*). Glass microelectrodes (~50 megohms) were advanced through the posterior end of the anteroventral cochlear nucleus using an LN Mini 55 micromanipulator (Luigs & Neumann, Germany), aiming toward the internal auditory canal. Acoustic stimulation was provided by an open-field Avisoft ScanSpeak Ultrasonic Speaker (Avisoft Bioacoustics, Germany). Noise bursts (50 ms) served as search stimuli. The spiking responses of isolated sound-responsive neurons were detected and recorded using a TDT system III hardware, amplified using an ELC-03XS amplifier (NPI Electronic, Tamm, Germany), and filtered using a bandpass filter (300 to 3000 Hz). Offline analysis was performed using waveform-based spike detection by a custom-written MATLAB script. Responses from the central SGN axons were identified and distinguished from cochlear nucleus neurons based on their stereotactic position, noise burst–induced firing, peristimulus time histogram (PSTH), regularity of firing, first spike latency, and spike waveform (*13*). The quality of the spikes was rated subjectively, and the recordings with a low signal-to-noise ratio were excluded from the analysis.

### Electron microscopy

#### Conventional embedding

Conventional embedding and transmission EM were performed as described before (*68*, *69*). Briefly, the cochleae of 1-month-old Ca_V_1.3^WT/WT^ and Ca_V_1.3^AG/AG^ mice (two animals per genotype) were fixed for 1 hour on ice with 4% paraformaldehyde and 0.5% glutaraldehyde in phosphate-buffered saline (pH 7.4). This was followed by the dissection of the apical turns of the organs of Corti and an additional overnight fixation with 2% glutaraldehyde in 0.1 M sodium cacodylate buffer (pH 7.2) at 4°C. Afterward, the samples were washed in 0.1 M sodium cacodylate buffer, followed by 1% osmium tetroxide treatment (v/v in 0.1 M sodium cacodylate buffer) for 1 hour. After an additional sodium cacodylate and distilled washing step, the samples were placed in 1% uranyl acetate for 1 hour for en bloc staining. Subsequently, the samples were washed three times in distilled water and dehydrated in a series of ascending concentrations of ethanol. Last, the samples were embedded in epoxy resin (Agar 100 kit, Plano, Germany) and polymerized for 48 hours at 70°C. Ultrathin sections (70 to 75 nm) were obtained from the polymerized blocks using a 35° diamond knife (Diatome AG, Biel, Switzerland) and an EM UC7 ultramicrotome (Leica Microsystems, Wetzlar, Germany). 2D electron micrographs were acquired at 80 kV using a JEM-1011 transmission EM (JEOL, Freising, Germany) equipped with a Gatan Orius SC1000 camera (Gatan, Munich, Germany).

#### Sample preparation for SBEM

For the SBEM experiment, cochlea samples were harvested from four Ca_V_1.3^WT/WT^ (M1, M2, M3, and M4) and two Ca_V_1.3^AG/AG^ (M5 and M6) mice at the ages of p36 to p68. The samples were chemically fixed and en bloc stained as previously described (*22*).

In short, the animals were decapitated after CO_2_ inhalation under anesthesia. The dissected cochleae were immediately perfused with an ice-cold fixative mixture through the round and oval windows using an infusion pump (Micro4, World Precision Instruments, Germany). The fixative solution was freshly prepared and contained 4% paraformaldehyde (Sigma-Aldrich, Germany) and 2.5% glutaraldehyde (Sigma-Aldrich, Germany) buffered with 0.08 M cacodylate (pH 7.4; Sigma-Aldrich, Germany). The samples were immersed in the fixative at 4°C for 5 hours and then transferred to a decalcifying solution made of the same fixative and 5% EDTA (SERVA, Germany) for another 5-hour incubation at 4°C. The samples were then washed twice with 0.15 M cacodylate (pH 7.4) for 30 min each and sequentially immersed in 2% OsO_4_ (Sigma-Aldrich, Germany), 2.5% ferrocyanide (Sigma-Aldrich, Germany), and 2% OsO_4_ at RT for 2, 2, and 1.5 hours, respectively. After being washed in 0.15 M cacodylate and distilled water (Sartorius, Germany) for 30 min each, the samples were sequentially incubated in filtered 1% thiocarbohydrazide (Sigma-Aldrich, Germany) solution and 2% OsO_4_ at RT for 1 and 2 hours, as well as in lead aspartate solution [0.03 M (pH 5.0); adjusted with KOH] at 50°C for 2 hours with two intermediate washing steps with distilled water at RT for 30 min each. The sample embedding was initiated with dehydration through graded precooled acetone (Carl Roth, Germany) series (50, 75, and 90% for 30 min each, all cooled at 4°C) and then pure acetone at RT (three times for 30 min each), followed by resin infiltration with 1:1 and 1:2 mixtures of acetone and Spurr’s resin monomer (4.1 g of ERL 4221, 0.95 g of DER 736, 5.9 g of NSA, and 1% DMAE; Sigma-Aldrich, Germany) at RT for 6 and 12 hours on a rotator. After being incubated in pure resin for 12 hours, the samples were placed in an embedding mold (PolyScience, Germany) and hardened in a prewarmed oven at 70°C for 72 hours.

#### Sample trimming and SBEM imaging

The sample blocks were mounted upright along the conical center axis on aluminum metal rivets (3VMRS12, Gatan, UK) and trimmed coronally toward the modiolus using a diamond trimmer (TRIM2, Leica, Germany). According to anatomical landmarks, block faces of about 600 mm by 800 mm with fields of view at the target segments were created using an ultramicrotome (UC7, Leica, Germany). Sample coating of a 30-nm-thick gold layer was done using a sputter coater (ACE600, Leica, Germany). The serial images were acquired using a field-emission scanning EM (Gemini300, Zeiss, Germany) equipped with an in-chamber ultramicrotome (3ViewXP, Gatan, UK) and backscattered electron detector (OnPoint, Gatan, UK). Focal charge compensation was set to 100% with a high vacuum chamber pressure of 2.8 × 10^3^ mbar. Nine datasets (M1/a, M2/a, M3/m, M4/m, M1/b, M2/b, M5/m, M6/m, and M5/b) were imaged at a 12-nm pixel size, and three datasets (M5/a, M6/a, and M6/b) were at an 11-nm pixel size for imaging. All datasets were acquired at 50-nm cutting thickness, 2-keV incident beam energy, and 1.5-ms pixel dwell time.

The Ca_V_1.3^WT/WT^ datasets comprised six image stacks, among which two were from the apical cochlear region (M1/a: 2377 slices with each of 9000 by 15,000 pixels and M2/a: 2048 slices of 6000 by 6000 pixels), two from mid-cochlea (M3/m: 3072 slices of 9000 by 14,000 pixels and M4/m: 2503 slices of 9000 by 15,000 pixels), and two from the basal cochlear region (M2/b: 3072 slices of 6000 by 6000 pixels and M1/b: 3080 slices of 6000 by 9000 pixels). The six stacks of Ca_V_1.3^AG/AG^ datasets contained 1024 slices (7000 by 9000 pixels, apex, M5/a), 2048 slices (5000 by 9000 pixels, apex, M6/a), 2864 slices (9000 by 15,000 pixels, mid, M5/m), 2048 slices (10,000 by 14,000 pixels, mid, M6/m), 3072 slices (7000 by 9000 pixels, base, M5/b), and 2699 slices (7000 by 9000 pixels, base, M6/b). All datasets were aligned along the *z* direction using a self-written MATLAB script based on cross-correlation maximum between consecutive slices (*70*) before being uploaded to webKnossos for data visualization and annotation.

#### Ribbon size measurement, synapse counting, and mitochondrial analysis

A total of 338 ribbon-type synapses were manually annotated in 24 intact IHCs captured by SBEM using webKnossos. The electron-dense region of individual ribbon synapses was manually contoured, and the associated voxels were counted for ribbon volume measurement. In the case of multi-ribbon synapses, all ribbon bodies at a single AZ were summed up to yield the ribbon volume. Sixty-seven large vesicle-free boutons were found to contact the IHC basal lateral poles, which were further identified as nonsynaptic terminals of SGN based on their characteristic neurite morphology (*71*). Mitochondrial analysis was performed as previously described (*71*).

### Data analysis

#### Ca^2+^ imaging with Fluo4-FF

Images were analyzed using Igor Pro Software (WaveMetrics). Ca^2+^ hotspots were identified by subtracting the average signal of several baseline frames from the average signal of five frames during stimulation (Δ*F* image). The intensities of the 3-by-3 matrix surrounding the central pixel of the hotspot were averaged across all time points to obtain the intensity profiles of Ca^2+^ influx over time. Afterward, the background signal, calculated as an average of approximately 60-by-60 pixel intensities outside the cell, was subtracted from the intensity-time profiles, and Δ*F*/*F*_0_ traces were calculated. The two Δ*F*/*F*_0_ traces (one shifted by 5 ms over the other) were combined, plotted against the corresponding voltages (*F*-*V* curves), and fitted with a modified Boltzmann function. Fractional activation curves were calculated by fitting the linear decay of the fluorescence signal from the *F*-*V* curves with a linear function (*G*_max_), dividing the *F*-*V* fit by the *G*_max_ line, and fitting the resulting curves with a Boltzmann function. Maximal Ca^2+^ influx (Δ*F*/*F*_0 max_) was calculated by averaging five points during the stimulation. The coordinates of the ribbons obtained from the fluorescence of TAMRA-conjugated Ctbp2 binding peptide were transferred from a Cartesian to a cylindrical coordinate system to assign the pillar and modiolar coordinates of the synapses, as previously described (*17*). Data were not considered for the position-dependent analysis of the AZs, whenever the morphology of the IHC was deformed.

#### iGluSnFR and Rhod-FF imaging

Images were analyzed using the Python software as described before (*18*). Briefly, the Δ*F* image for all depolarizations was calculated by subtracting the average of 10 baseline images right before the stimulation from the average of five frames during the stimulation. To detect the regions of interest (ROIs), the average projection of Δ*F* images from all depolarizations was median filtered (filter level: 0.5 to 4) and maximum entropy thresholding was applied. Touching boutons were separated by watershed segmentation. To ensure that the signal at the ROIs originated from a single AZ, only regions that had a corresponding single Ca^2+^ hotspot, or those adequately spaced apart from each other, were analyzed further. The average of all pixels of each ROI was calculated at all time points. Afterward, the background signal (average of approximately 60-by-60 pixel intensities outside the cell) was subtracted from the intensity-time profiles, and Δ*F*/*F*_0_ traces were calculated. Peak detection was performed by smoothing the Δ*F*/*F*_0_ traces using the Hanning window function (window size: 7). For the area under the curve (AUC) calculation, the initial segment of Δ*F*/*F*_0_ traces before stimulation was fitted with an exponential function, and the resulting fit was subtracted from Δ*F*/*F*_0_ to accommodate for photobleaching. Subsequently, the AUC between stimulation and 20 frames poststimulation was computed. AUCs of different depolarizations were plotted against voltages, normalized, and fitted with the Boltzmann function to obtain the threshold of glutamate release defined as 10% of maximal release (*V*_10_), voltage of half-maximal glutamate release (*V*_half_), and dynamic range of release, defined as the voltage range between 10 and 90% of maximal release.

Δ*F* images of the Rhod-FF signal were calculated by subtracting the average of baseline frames from the average of five frames during the highest stimulation at each of the five planes. Δ*F* images of five planes were averaged to visualize the hotspots. ROI detection was done similar to iGluSnFR ROI detection. The average of all pixels of each ROI was calculated at all time points for all five planes. The plane with the highest signal intensity was used to calculate Δ*F*/*F*_0_. A band-stop filter was applied to Δ*F*/*F*_0_ traces to remove 33-Hz noise caused by the spinning disk. Two Δ*F*/*F*_0_ traces corresponding to two identical voltage ramp depolarizations were averaged and plotted against voltage. The modified Boltzmann function was fitted to normalized *F*-*V* curves similar to the ones obtained from Fluo4-FF imaging. Voltage of half-maximal Ca^2+^ influx (*V*_half_) and dynamic range, defined as the voltage range between 10 and 90% of maximal Ca^2+^ influx, were calculated from modified Boltzmann fits. Maximal Ca^2+^ influx (Δ*F*/*F*_0 max_) was calculated by averaging three points during the stimulation. Ca^2+^ influx–release coupling at IHC single synapses was estimated by relating the Boltzmann fits of normalized glutamate release to the normalized Ca^2+^ influx of matching voltage ranges and fitting the initial 25% of the glutamate release with a power function. Data were discarded from further analysis if the fluorescence signal of the individual synapses could not be well separated from one another and the voltage dependence of iGluSnFR and Rhod-FF fluorescence could not be reliably fitted with a Boltzmann function (goodness of fit: < 0.7).

#### Immunofluorescence analysis

The Ctbp2 and Ca_V_1.3 immunofluorescent puncta in confocal stacks were analyzed using the Imaris software (version 9.6.0; Bitplane) automatic spot detection algorithm. The intensities of the ribbons and Ca_V_1.3 clusters were calculated by summating pixel intensities of the 7-by-7-by-5 region around the center of mass of each immunofluorescent spot. Spatial gradients of the ribbon and Ca_V_1.3 cluster sizes were analyzed using Imaris and custom-written MATLAB scripts, as described before (*33*, *72*, *73*). Data were excluded from the position-dependent analysis, whenever the morphology of the IHCs was deformed.

2D STED images of Ca_V_1.3 line-like clusters were analyzed using the Igor Pro software. Briefly, the 2D Gaussian function was fitted to the clusters to obtain full width at half maxima of long and short axes using a genetic fit algorithm (*74*). The brightness and contrast of the representative images were adjusted for visualization using the Fiji software.

#### Statistical analysis

Data were analyzed using the Igor Pro (WaveMetrics), Python, and MATLAB software. For two-sample comparisons, data were tested for normality and equality of variances using Jarque-Bera and *F* tests, respectively. Afterward, two-tailed *t* test or Wilcoxon rank sum test was performed. The latter was used when normality and/or equality of variances were not met. For multiple comparisons, one-way analysis of variance (ANOVA) followed by Tukey’s post hoc multiple comparison test (for normally distributed data) or Kruskal-Wallis followed by Dunn’s multiple comparison test (for nonnormally distributed data) was used. *P* values were corrected for multiple comparisons using the Holm-Šídák (for Dunn’s test) or Bonferroni-Holm (for Fig. 4C) method. Data are presented as mean ± SEM, unless otherwise stated. The number of animals is reported as *N*.

## Supplementary Materials

### This PDF file includes:

Supplementary Text Figs. S1 to 12 Table S1 References
