# Supplementary material for: Gating of hair cell Ca2+ channels governs the activity of cochlear neurons
Source: Sci Adv. 2025 Jun 18;11(25):eadu7898. doi: 10.1126/sciadv.adu7898 (PMC12225641; doi:10.1126/sciadv.adu7898)
Supplement: Supplementary file 1 — Supplementary Text Figs. S1 to 12 Table S1 References [file sciadv.adu7898_sm.pdf]

Science Advances
AAAS

Supplementary Materials for

Gating of hair cell Ca^{2+} channels governs the activity of cochlear neurons

Nare Karagulyan *et al.*

Corresponding author: Tobias Moser, tmoser@gwdg.de

Sci. Adv. **11**, eadu7898 (2025)
DOI: 10.1126/sciadv.adu7898

This PDF file includes:

Supplementary Text
Figs. S1 to 12
Table S1
References

Supplementary Text

IHC-SGN synaptic transmission model

IHC-SGN synaptic transmission and SGN spike generation was modeled according to Meddis et al., 1990 (*58*) using Igor Pro software. In brief, the transmitter release rate/fraction [k(t)], is a sigmoidal function directly dependent on the stimulus level [st(t)]: $k(t)=g(st(t) + A) / (st(t) + A + B)$. The parameter g represents the maximal release rate, A controls the baseline release, as well as the threshold of the release and B controls the saturation and the steepness of the curve. Under the assumption of Ca^{2+} nanodomain control of release (Fig. 4), k(t) reflects the stimulus dependence of Ca^{2+} channel activation. The parameters A and B of the release fraction equation are modified in order to shift the sigmoidal function (Table S1, fig. S12). The release fraction determines the synaptic cleft transmitter content (c). Spike generation is scaled by the cleft transmitter content, firing probability scaling factor (h) and further modulated by absolute (0.8 ms) and relative (2 ms) refractory periods (*75*). The relative refractory period is a random number drawn from a monoexponential distribution with an average value of 2 ms. Rate-level functions are obtained by calculating the adapted firing rates from the simulated PSTH (50 ms stimulation duration).

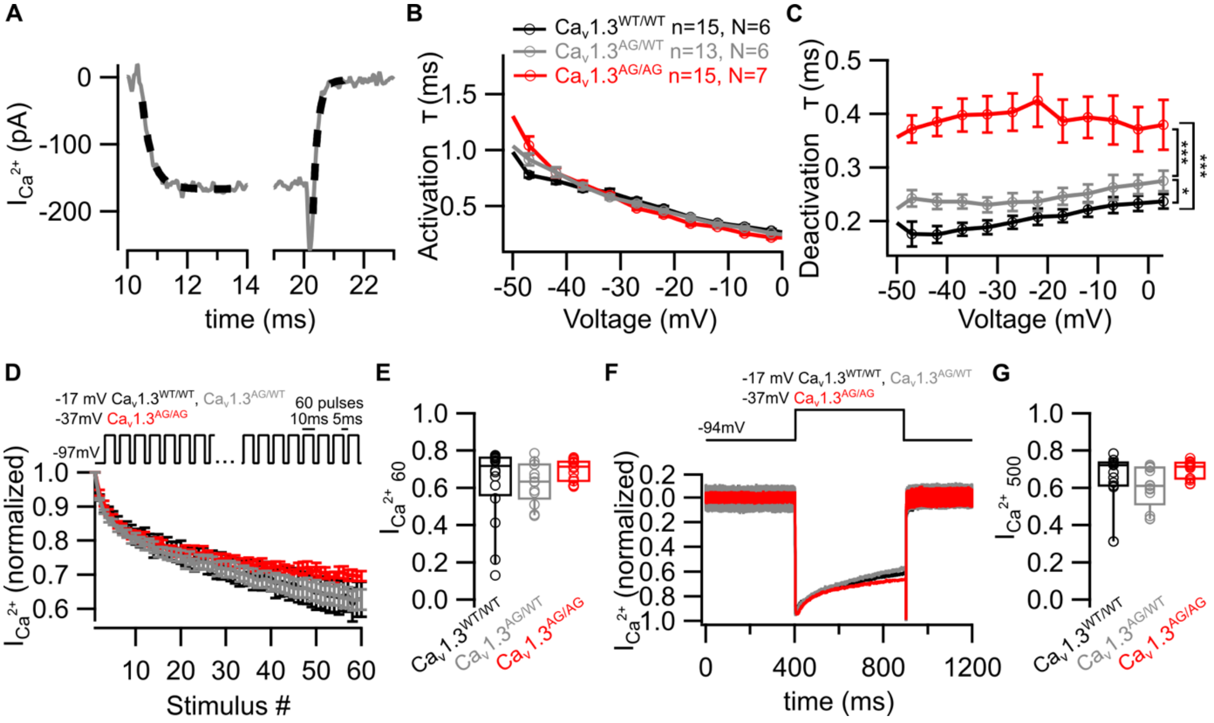

Fig. S1.

Slow deactivation of Ca^{2+} channels in IHCs of Cav1.3$^{AG/WT}$ and Cav1.3$^{AG/AG}$ mice. (**A**) Activation and deactivation constants of Ca^{2+} currents were obtained by fitting exponential functions (dotted lines) to the first 3 ms of activation and 1 ms of deactivation. (**B**) Cav1.3 activation kinetics across voltages are not changed in IHCs of Cav1.3$^{AG/WT}$ and Cav1.3$^{AG/AG}$ mice. (**C**) The deactivation kinetics are slower in IHCs of Cav1.3$^{AG/WT}$ and Cav1.3$^{AG/AG}$ mice. (**D**) Normalized and averaged Cav1.3 currents measured by applying 60 repetitive stimulations with 10 ms duration and 5 ms interstimulus interval in IHCs of Cav1.3$^{WT/WT}$, Cav1.3$^{AG/WT}$, and Cav1.3$^{AG/AG}$ mice. Error bars show ± SEM. (**E**) The fraction of Ca^{2+} current remaining after 60 repetitive stimulations is comparable in Cav1.3$^{WT/WT}$, Cav1.3$^{AG/WT}$, and Cav1.3$^{AG/AG}$ IHCs. (**F**) Representative normalized Ca^{2+} currents measured by applying 500 ms depolarization at the maximal activation voltage in Cav1.3$^{WT/WT}$, Cav1.3$^{AG/WT}$, and Cav1.3$^{AG/AG}$ IHCs. (**G**) The fraction of Ca^{2+} current remaining after 500 ms depolarization is comparable in Cav1.3$^{WT/WT}$, Cav1.3$^{AG/WT}$, and Cav1.3$^{AG/AG}$ IHCs. Data in (B), (C) and (D) is presented as mean ± SEM. Box-Whisker plots with individual data points overlaid show median, 25th and 75th percentiles (box), 10th and 90th percentiles (whiskers). Statistical significances were determined using one-way ANOVA/Kruskal-Wallis test followed by Tukey's HSD/Dunn's test for each voltage for (B) and (C) and Kruskal-Wallis test for (E) and (G). Significances are reported as *$p < 0.05$, ***$p < 0.001$.

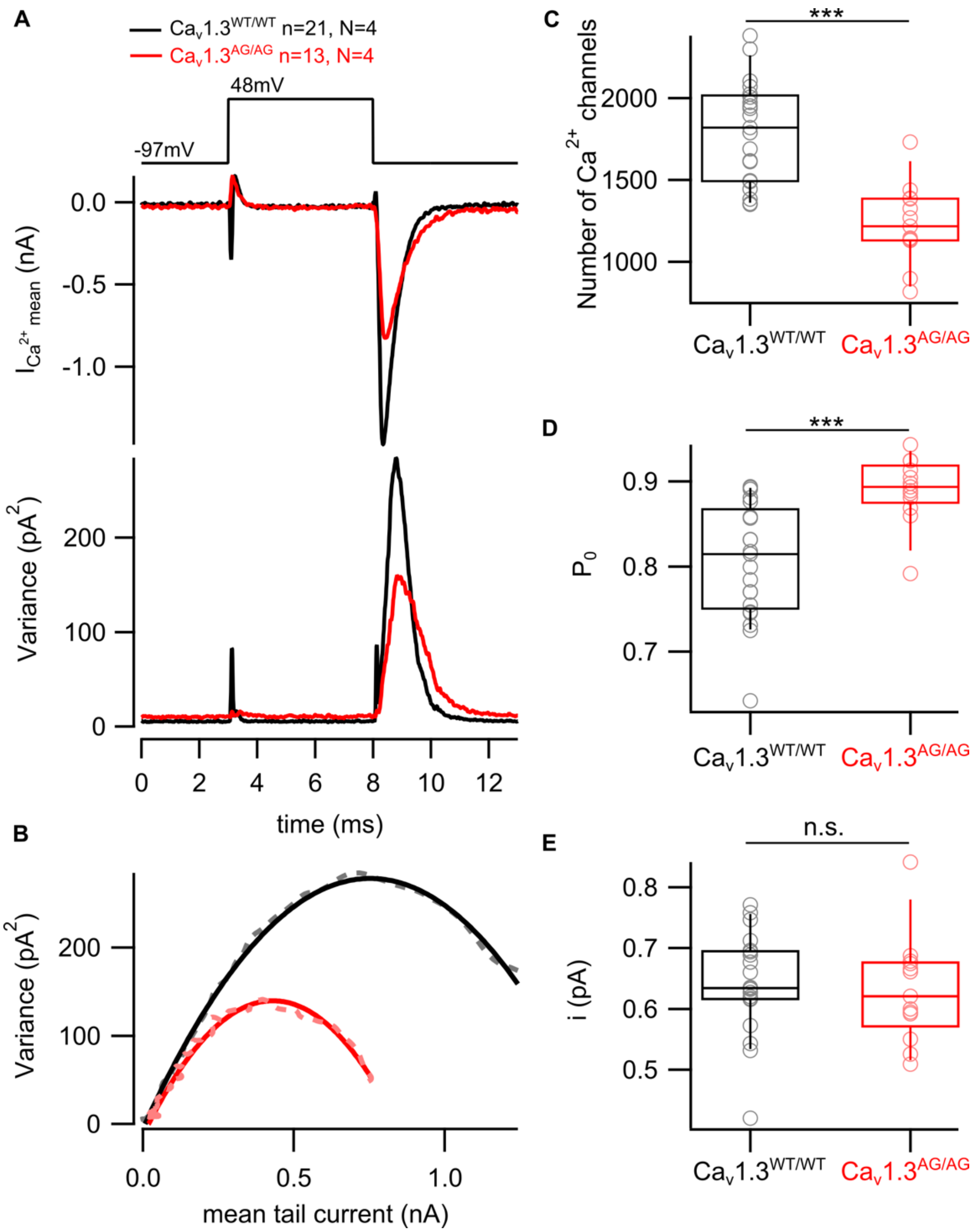

Fig. S2.

Increased open probability and decreased number of Ca_V1.3 channels in $Ca_V1.3^{AG/AG}$ IHCs. (**A**) Exemplary mean Ca^{2+} current (middle) evoked by the voltage clamp protocol (top) and variance of the mean current (bottom). Currents were recorded in the presence of 5 µM BayK and 10 mM extracellular Ca^{2+}. (**B**) Data from exemplary cells showing variance of the mean Ca^{2+} current plotted against the mean Ca^{2+} current and fitted with a quadratic function. (**C**) The number of activatable Ca^{2+} channels is reduced in $Cav1.3^{AG/AG}$ IHCs. (**D**) Ca^{2+} channels in $Cav1.3^{AG/AG}$ IHCs show higher open probability (P_o) compared to $Cav1.3^{WT/WT}$ IHCs. (**E**) Single channel current (i) of Ca^{2+} channels is not changed in IHCs of $Cav1.3^{AG/AG}$ mice. Box-Whisker plots with individual data points overlaid show median, 25th and 75th percentiles (box), 10th and 90th percentiles (whiskers). Statistical significances were determined using two-tailed Wilcoxon

rank-sum test for (C), two-tailed t-test for (D) and (E). Significances are reported as ***$p < 0.001$.

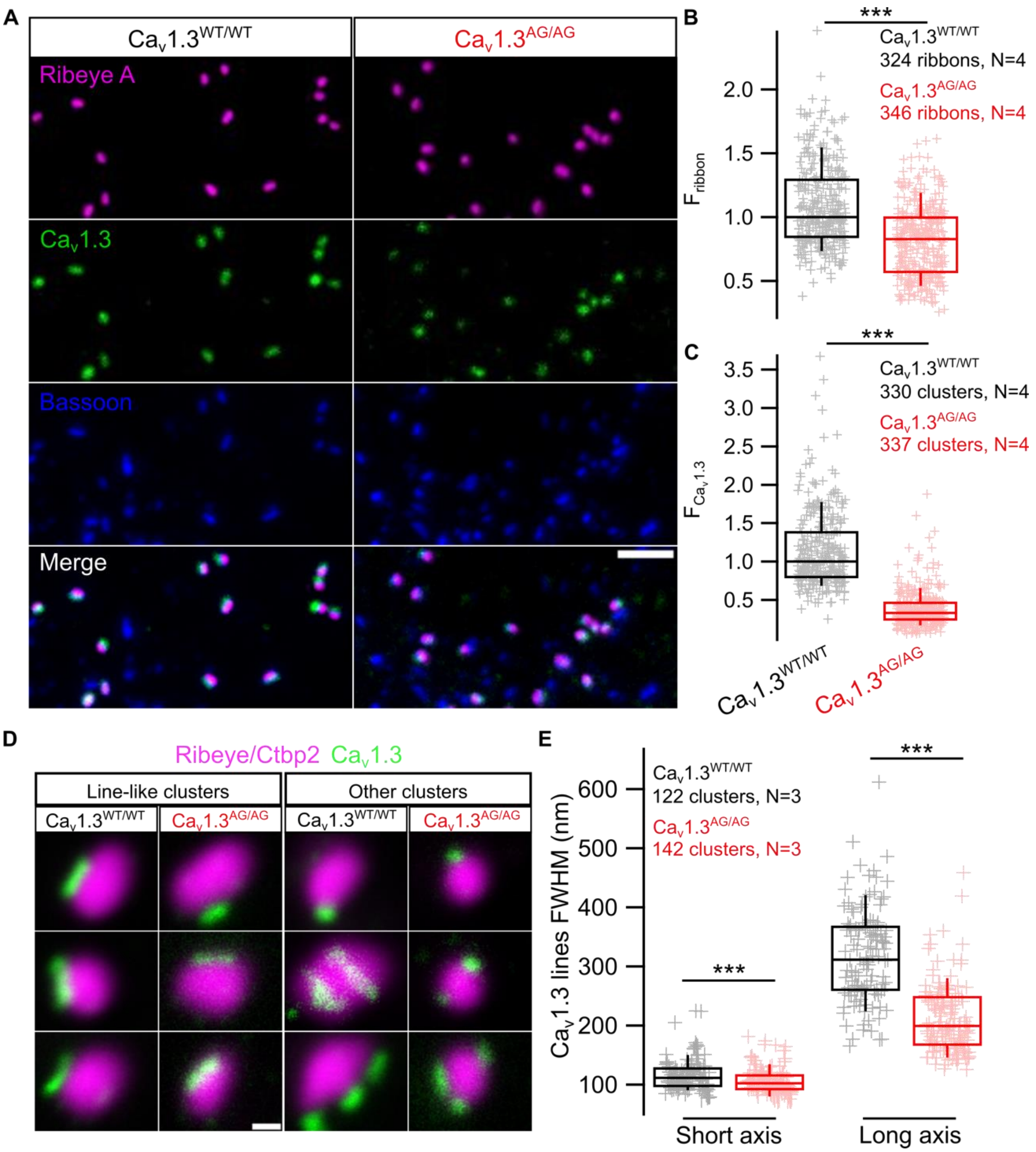

Fig. S3.

Smaller Cav1.3 channel clusters and ribbons at active zones of apical Cav1.3$^{AG/AG}$ IHCs.
(**A**) Maximum intensity projections of confocal stacks acquired from IHC synaptic regions and immunolabeled against Ribeye A, Bassoon and Cav1.3. Scale bar = 2 µm. (**B**) Immunofluorescence intensity of the synaptic ribbons at the apical turn of the cochlea is reduced in Cav1.3$^{AG/AG}$ IHCs. All values were normalized to the median intensity of the Cav1.3$^{WT/WT}$ ribbons. (**C**) Immunofluorescence intensity of synaptic Cav1.3 positive puncta obtained from the confocal stacks is reduced in Cav1.3$^{AG/AG}$ IHCs. All values were normalized to the median intensity of the Cav1.3$^{WT/WT}$ channel clusters. (**D**) Representative images of IHC AZs acquired by STED imaging of AZs immunolabeled for Ribeye/Ctbp2 and Cav1.3. Scale bar = 200 nm. (**E**) Cav1.3 line-like clusters fitted with 2D gaussian function show reduced full with at half maxima

(FWHM) of long and short axes in $Cav1.3^{AG/AG}$ IHCs. Box-Whisker plots with individual data points overlaid show median, 25th and 75th percentiles (box), 10th and 90th percentiles (whiskers). Statistical significances were determined using two-tailed Wilcoxon rank-sum test for data in (B), (C) and (E). Significances are reported as $***p < 0.001$.

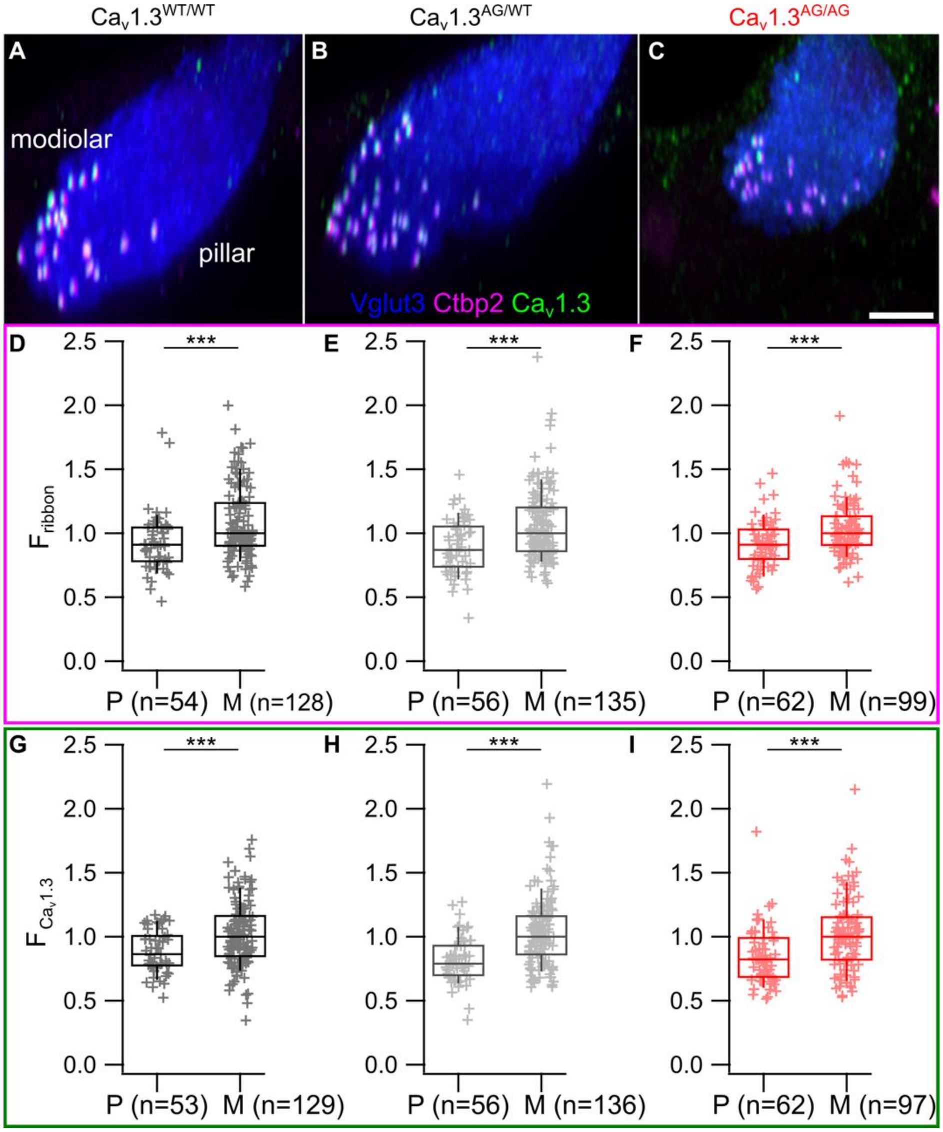

Fig. S4.

Modiolar-pillar gradients of ribbon size and Ca$_V$1.3 cluster size are preserved in apical IHCs of Ca$_V$1.3$^{AG/WT}$ and Ca$_V$1.3$^{AG/AG}$ mice. (**A-C**) Maximal intensity projections of approximately 2 IHCs from Cav1.3$^{WT/WT}$ (A), Cav1.3$^{AG/WT}$ (B), Cav1.3$^{AG/AG}$ (C) mice immunolabeled against Vglut3, Ctbp2, Cav1.3. Scale bar = 5 µm. (**D-F**) Comparison of the immunofluorescence intensities of pillar and modiolar ribbons in IHCs of Cav1.3$^{WT/WT}$ (D), Cav1.3$^{AG/WT}$ (E), Cav1.3$^{AG/AG}$ (F) mice. (**G-I**) Comparison of immunofluorescence intensities of pillar and modiolar Cav1.3 clusters in IHCs of Cav1.3$^{WT/WT}$ (G), Cav1.3$^{AG/WT}$ (H), Cav1.3$^{AG/AG}$ (I) mice. Data were acquired from N = 2 (Cav1.3$^{WT/WT}$), 1 (Cav1.3$^{AG/WT}$), 2 (Cav1.3$^{AG/AG}$) mice. Box-Whisker plots with individual data points overlaid show median, 25th and 75th percentiles (box), 10th and 90th percentiles (whiskers). Statistical significances were determined using two-tailed Wilcoxon rank-sum test for data in (D-I). Significances are reported as ***$p < 0.001$.

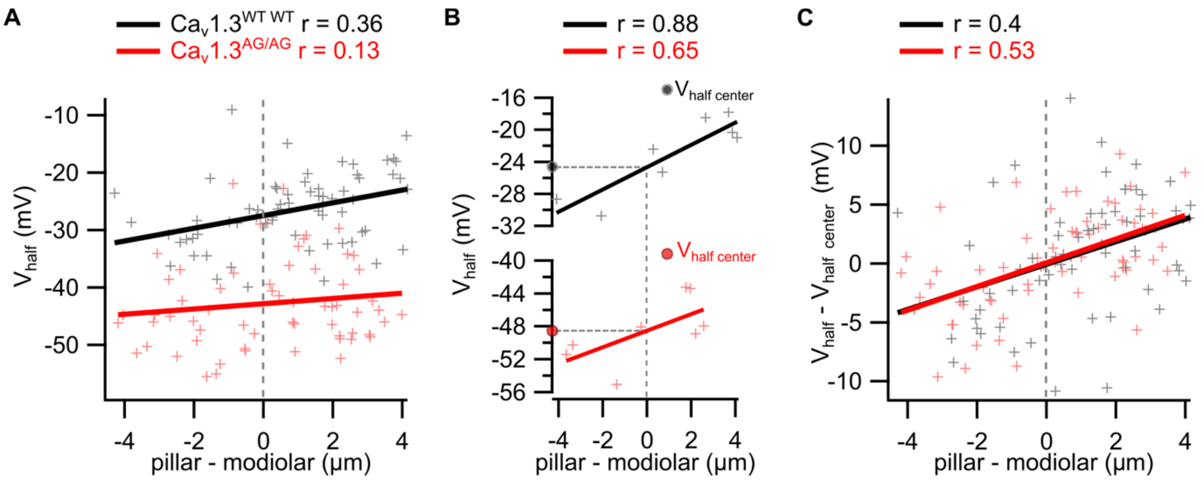

Fig. S5.

Individual IHCs show a pillar-modiolar gradient of voltage of half maximal activation. (**A**) V_{half} of Ca^{2+} channels at single AZs plotted against their position along the pillar-modiolar axis of the IHC. Thick lines show linear regression lines. Dotted lines indicate the center of the pillar-modiolar axis. (**B**) Same as (A) but from 2 representative cells. $V_{half\ center}$ obtained from the linear fit of V_{half} vs pillar-modiolar position, shows predicted V_{half} of each cell at the center of pillar-modiolar axis. (**C**) $V_{half\ center}$ of each IHC was subtracted from V_{half} of each AZ obtained from the cell, afterwards the data from all the recorded cells were pooled and fitted with linear function. Pearson's correlation coefficient is shown as (r).

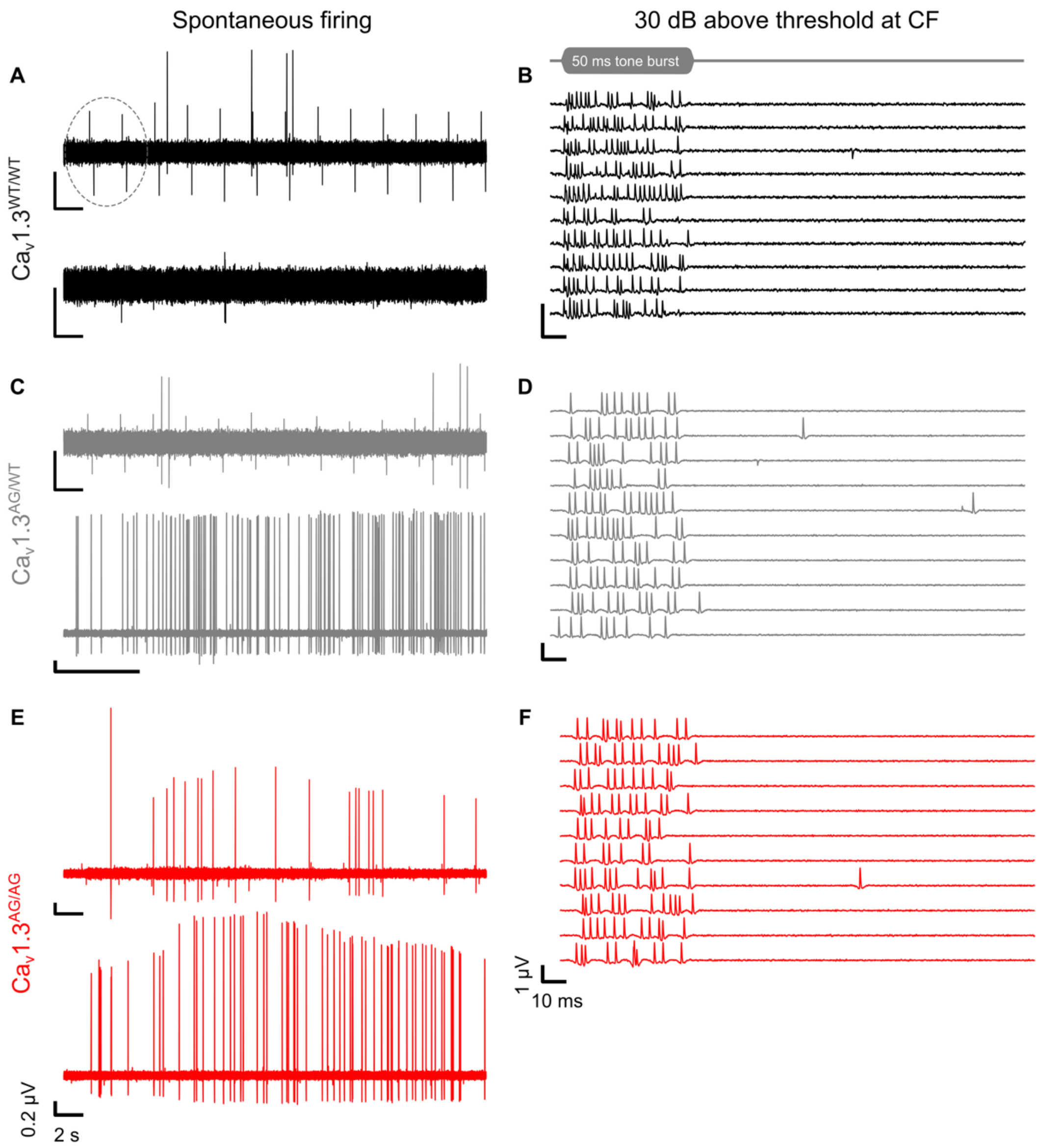

Fig. S6.

Exemplary recordings of spontaneous and evoked spiral ganglion neuron activity. Exemplary raw data traces of spontaneous and evoked firing recorded from single putative SGNs in Cav1.3$^{WT/WT}$ (A, B), Cav1.3$^{AG/WT}$ (C, D) and Cav1.3$^{AG/AG}$ (E, F) mice. Spontaneous firing from 2 different units it provided for each genotype. Positive and negative transients appearing in some of the spontaneous responses (shown with a dotted circle on the exemplary Cav1.3$^{WT/WT}$ trace) at about 0.43 Hz were an artifact of our heating plate and were removed by filtering prior to the analysis of spontaneous spike rate.

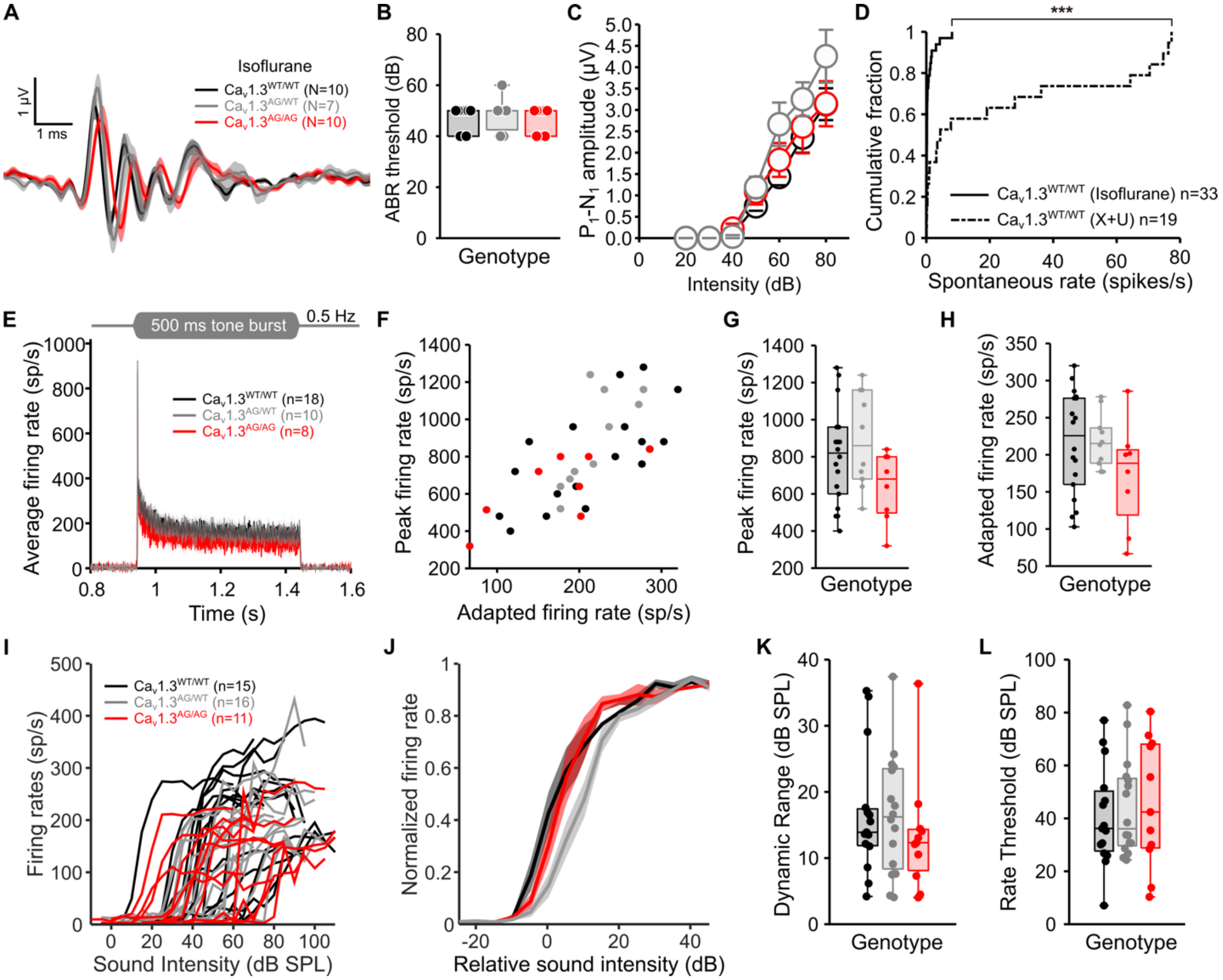

Fig. S7.

Comparable dynamic range of sound encoding by SGNs of Cav1.3$^{WT/WT}$, Cav1.3$^{AG/WT}$ and Cav1.3$^{AG/AG}$ mice. (**A**) Average ABR waveforms in response to 80 dB clicks recorded in mice under isoflurane anesthesia. (**B**) ABR thresholds in response to click stimuli are comparable in Cav1.3$^{WT/WT}$, Cav1.3$^{AG/WT}$ and Cav1.3$^{AG/AG}$ mice. (**C**) P$_1$-N$_1$ amplitude across different sound levels are comparable in Cav1.3$^{WT/WT}$, Cav1.3$^{AG/WT}$ and Cav1.3$^{AG/AG}$ mice. (**D**) SRs of SGNs recorded from Cav1.3$^{WT/WT}$ mice under isoflurane anesthesia are lower than those recorded under urethane/xylazine aesthesia. (**E**) Average PSTH in response to 500 ms tone burst stimulation at the CF, 30 dB above the threshold level and 0.5 Hz stimulation rate. Shaded areas show ± SEM. (**F, G** and **H**) Onset (G) and adapted (H) firing rates calculated from PSTH in (E) are not changed in Cav1.3$^{AG/WT}$ and Cav1.3$^{AG/AG}$ mice. (**I**) Rate level functions (RLFs) of individual SGNs recorded in response to 50 ms stimulation at the CF, 30 dB above the threshold level and stimulation rate of 5 Hz. (**J**) Average and normalized RLFs of SGNs, whereby the RLF of each SGN was further adjusted relative to its threshold (determined from the RLF). Shaded areas show ± SEM. (**K** and **L**) dynamic ranges (K) and the thresholds (L) calculated from RLFs are not changed in Cav1.3$^{AG/WT}$ and Cav1.3$^{AG/AG}$ mice. Single unit recordings were obtained from N = 6 (Cav1.3$^{WT/WT}$), 3 (Cav1.3$^{AG/WT}$), 6 (Cav1.3$^{AG/AG}$) mice. Box-Whisker plots with individual data points overlaid show median, 25th and 75th percentiles (box), and the range (whiskers). Statistical

significances were determined using Kruskal-Wallis test for (B), (G), (H), (K) and (L), Kruskal-Wallis test followed by Tukey-Kramer multiple comparison test for each sound level for (C), Kolmogorov-Smirnov and two-tailed Wilcoxon rank-sum test for (D). Significances are reported as ***$p < 0.001$.

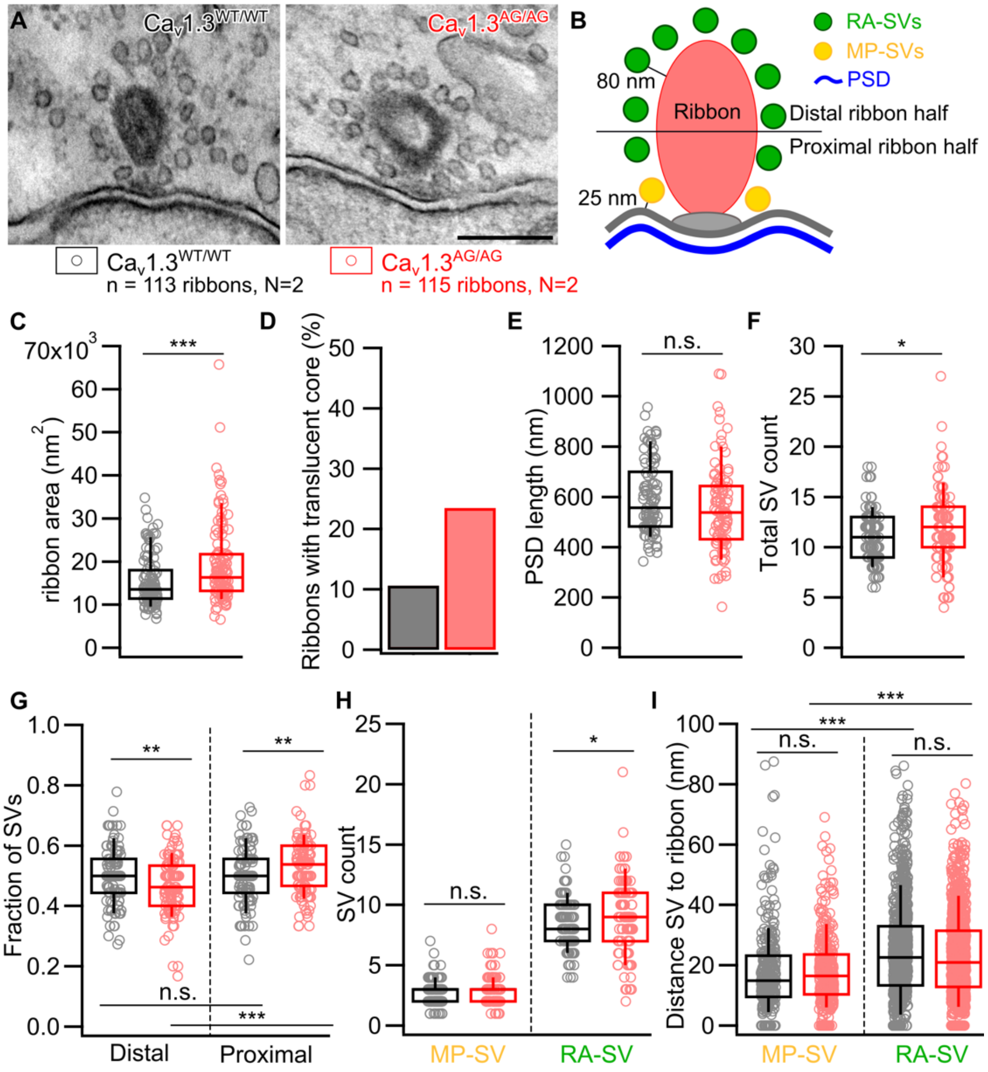

Fig. S8.

Larger fraction of ribbons with hollow cores in Cav1.3$^{AG/AG}$ IHCs. (**A**) Representative electron micrographs of the ribbon synapses from Cav1.3$^{WT/WT}$ and Cav1.3$^{AG/AG}$ IHCs. (**B**) Schematic illustration of the quantitative analysis of random EM sections. (**C**) Increased ribbon area in Cav1.3$^{AG/AG}$ IHCs. (**D**) Higher percentage of ribbons with translucent core in Cav1.3$^{AG/AG}$ IHCs. (**E**) The length of the postsynaptic density (PSD) is not changed at the afferent synapses of Cav1.3$^{AG/AG}$ IHCs. (**F**) Total vesicle number associated with the ribbons is increased in Cav1.3$^{AG/AG}$ IHCs. (**G**) The fraction of the vesicles associated with the distal half of the ribbon (away from the plasma membrane) is decreased, while those associated with the proximal ribbon half (close to the plasma membrane) is increased in Cav1.3$^{AG/AG}$ IHCs. (**H**) The number of the membrane proximal synaptic vesicles is unchanged, while the number of the ribbon associated vesicles is increased in Cav1.3$^{AG/AG}$ IHCs. (**I**) The distance of the membrane proximal and ribbon associated vesicles from the ribbon is not changed in Cav1.3$^{AG/AG}$ IHCs. Each genotype represents data from N = 2 mice. Box-Whisker plots with individual data points overlaid show median, 25th and 75th percentiles (box), 10th and 90th percentiles (whiskers). Statistical significances were determined using two-tailed Wilcoxon rank-sum test for (C), (E), (F), (H) and

(I) and two-tailed t-test for (G)**.** Significances are reported as *$p < 0.05$, **$p < 0.01$, ***$p < 0.001$.

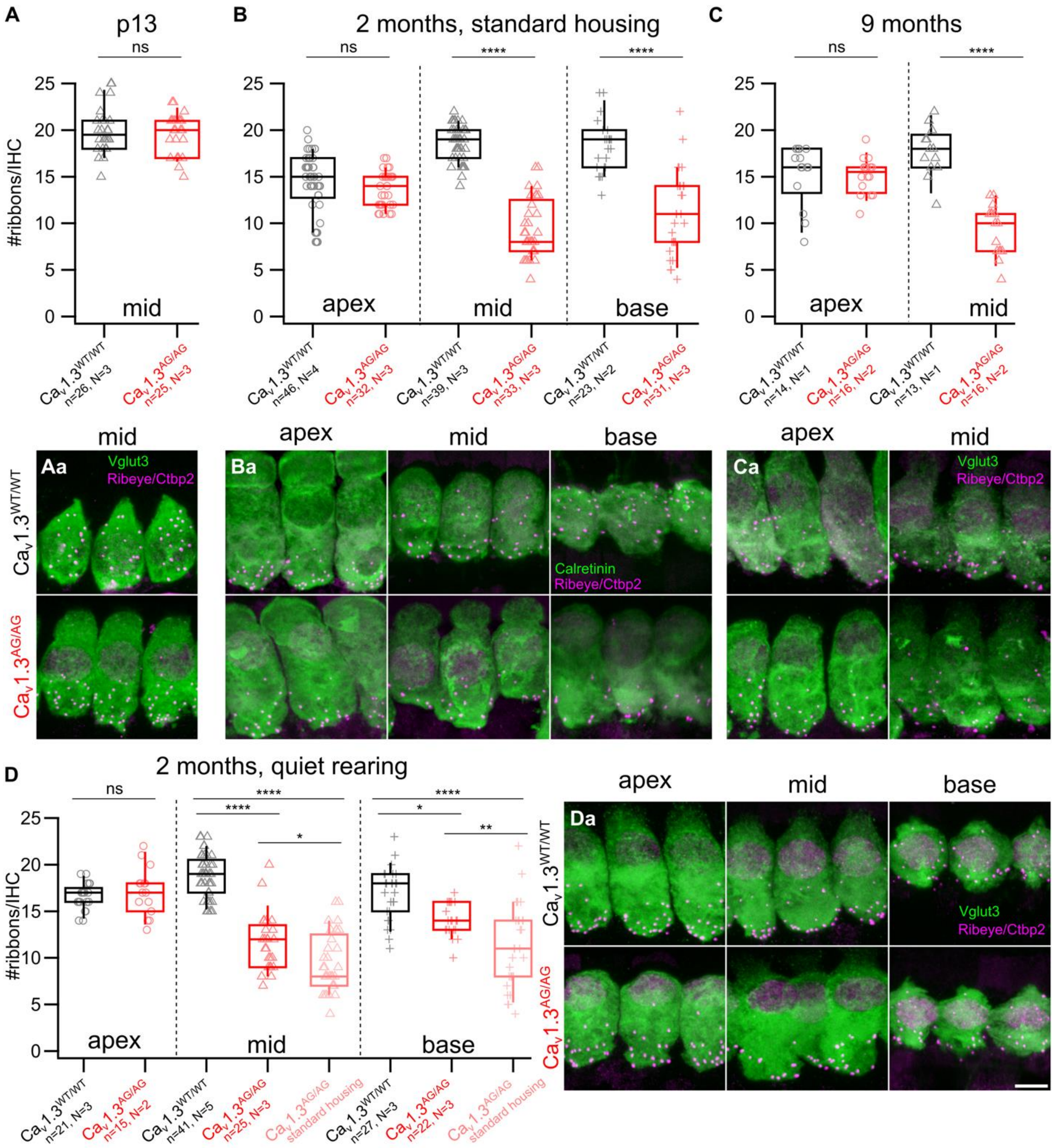

Fig. S9.

$Ca_V1.3^{AG/AG}$ IHCs exhibit loss of ribbons at the middle and basal turns of the cochlea after the hearing onset. (**A-D**) Number of the ribbons at different tonotopic locations was counted in the confocal stacks of IHCs of p13 (A), 2-month-old (B), 9-month-old (C) and 2-month-old quietly reared (D) mice. (**Aa-Da**) Maximal intensity projections of the representative IHCs immunolabeled against Ribeye/Ctbp2 and calretinin or Vglut3. Scale bar = 5 µm. Box-Whisker plots with individual data points overlaid show median, 25th and 50th quartiles (box), 10th and 90th percentiles (whiskers). Statistical significances were determined using two-tailed Wilcoxon

rank-sum test for each tonotopic location followed by Bonferroni-Holm multiple comparison correction for (A), (B) and (C) and Kruskal-Wallis followed by Dunn's multiple comparison test for middle and basal cochlear regions for (D). Significances are reported as *$p < 0.05$, **$p < 0.01$, ****$p < 0.0001$.

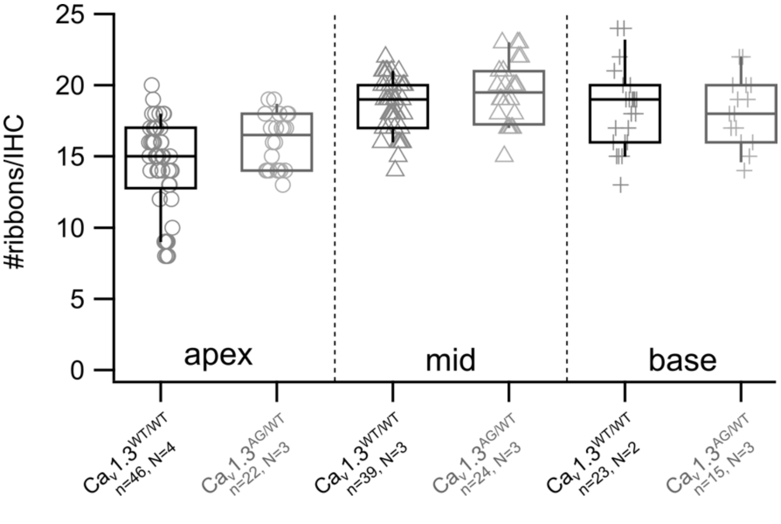

Fig. S10.

The number of the synaptic ribbons remains unchanged in IHCs of $Ca_V1.3^{AG/WT}$ mice. Quantification of the ribbon number in 2-month-old mice was performed in confocal stacks of IHCs immunolabeled against Vglut3 and Ctbp2. Box-Whisker plots with individual data points overlaid show median, 25th and 75th percentiles (box), and the range (whiskers).

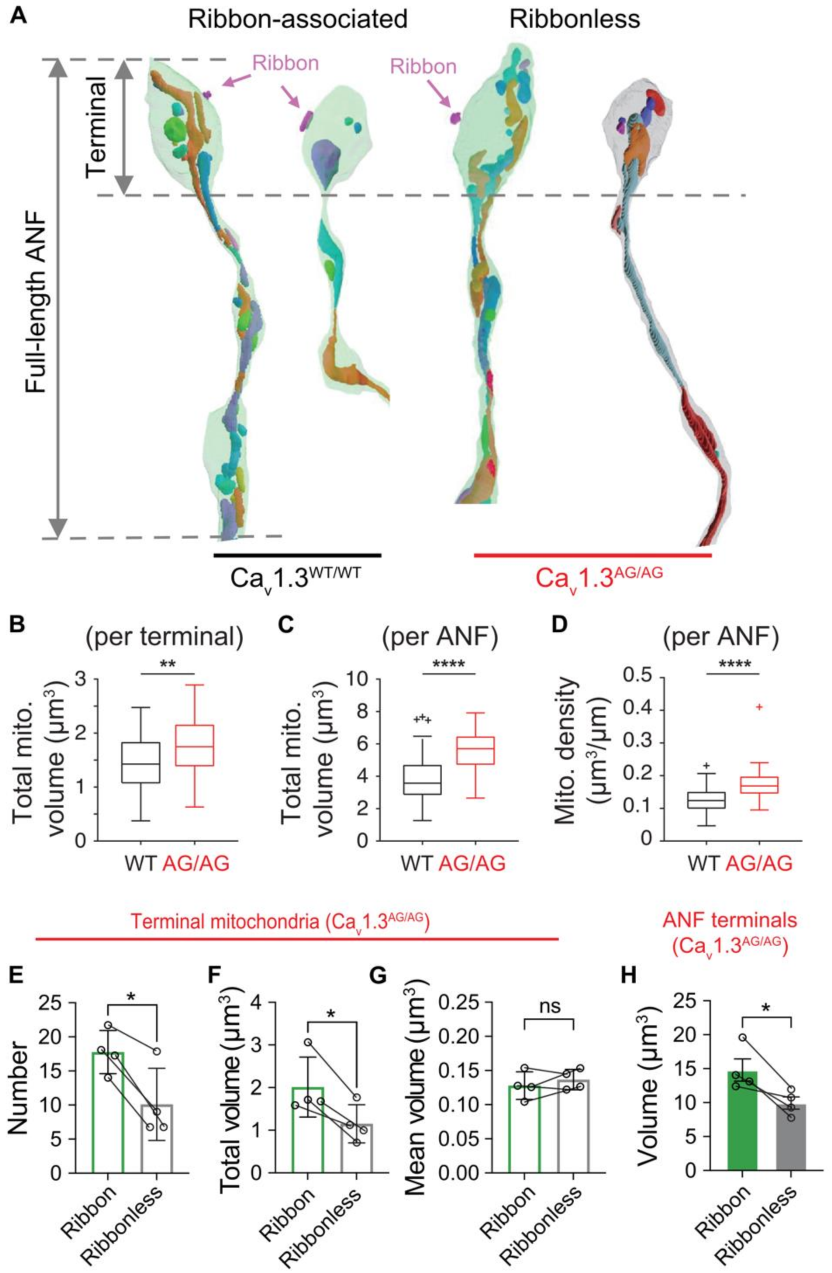

Fig. S11.

Quantification of SGN mitochondria content in SBEM reconstructions of Ca$_V$1.3$^{WT/WT}$ and Ca$_V$1.3$^{AG/AG}$ mid-cochlear region. (**A**) Mitochondrial reconstructions of example auditory nerve fiber (ANF, peripheral neurite of SGN) postsynaptic to the Cav1.3$^{WT/WT}$ (left) and Cav1.3$^{AG/AG}$ (right) IHCs. (**B** and **C**) In contrast to Cav1.3$^{WT/WT}$ (black), total mitochondrial volumes are larger in both terminals (B) and full-length peripheral neurites (C) of ribbon-associated SGNs in Cav1.3$^{AG/AG}$ (red) cochlea. (**D**) Higher ANF mitochondrial density of Cav1.3$^{AG/AG}$ (red) than that of Cav1.3$^{WT/WT}$ cochlea (black). (**E** and **F**) For SGNs on Cav1.3$^{AG/AG}$ IHCs, the ribbon-associated terminal (green) features a greater number and larger total volume of mitochondria than those without a ribbon (grey). (**G**) Mean sizes of mitochondria are comparable between

ribbon-associated and ribbonless terminals. (**H**) Ribbon-associated SGNs (green) have a larger terminal size than ribbonless SGNs (grey). Each genotype represents data from N = 2 mice. Statistical significances were determined using two-tailed t-test for (B), (C) and (D) and paired t-test for (E), (F), (G) and (H). Significances are reported as $^{*}p < 0.05$, $^{**}p < 0.01$, and $^{****}p < 0.0001$.

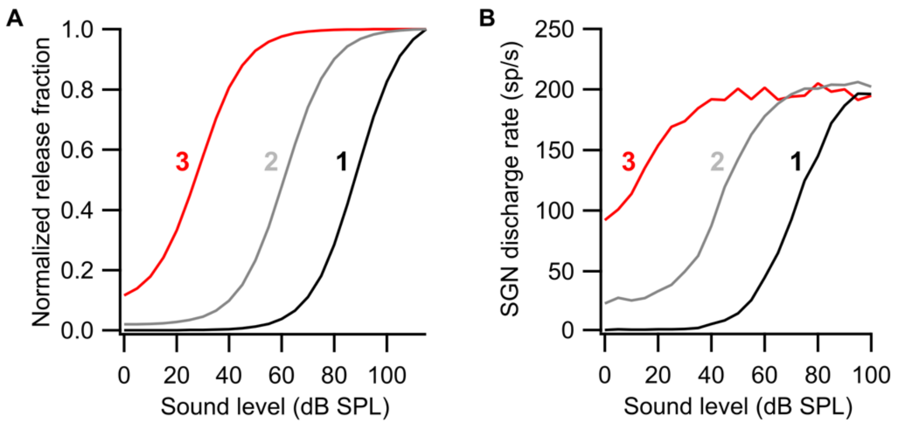

Fig. S12.

The effects of modifying model parameters A and B on the SGN rate-level function. (**A** and **B**) Normalized release fractions (A) and their corresponding rate-level functions (B). The exact parameters can be found in Table S1.

Table S1.

Model parameters. Parameters A and B of the release fraction equation were modified, but the rest of the parameters were kept constant.

**Parameters**	**1**	**2**	**3**
A	1	1	0.1
B	1200	50	1.1
g	1660	1660	1660
dt (s)	0.0001	0.0001	0.0001
y (replenishment rate)	16.6	16.6	16.6
l (rate of loss from the cleft)	500	500	500
r (rate of return from the cleft)	12500	12500	12500
x (rate of release from reprocessing to free transmitter)	3000	3000	3000
h (firing probability scaling factor)	10000	10000	10000

REFERENCES AND NOTES

1. T. Moser, N. Karagulyan, J. Neef, L. M. Jaime Tobón, Diversity matters—Extending sound intensity coding by inner hair cells via heterogeneous synapses. *EMBO J.* **42**, e114587 (2023).

2. M. A. Rutherford, H. Von Gersdorff, J. D. Goutman, Encoding sound in the cochlea: From receptor potential to afferent discharge. *J. Physiol.* **599**, 2527–2557 (2021).

3. B. R. Shrestha, L. V. Goodrich, "Wiring the cochlea for sound perception" in *The Oxford Handbook of the Auditory Brainstem,* K. Kandler, Ed. (Oxford Univ. Press, 2019).

4. J. Platzer, J. Engel, A. Schrott-Fischer, K. Stephan, S. Bova, H. Chen, H. Zheng, J. Striessnig, Congenital deafness and sinoatrial node dysfunction in mice lacking class D L-type Ca^{2+} channels. *Cell* **102**, 89–97 (2000).

5. A. Brandt, J. Striessnig, T. Moser, $Ca_V1.3$ channels are essential for development and presynaptic activity of cochlear inner hair cells. *J. Neurosci.* **23**, 10832–10840 (2003).

6. S. M. Baig, A. Koschak, A. Lieb, M. Gebhart, C. Dafinger, G. Nürnberg, A. Ali, I. Ahmad, M. J. Sinnegger-Brauns, N. Brandt, J. Engel, M. E. Mangoni, M. Farooq, H. U. Khan, P. Nürnberg, J. Striessnig, H. J. Bolz, Loss of $Ca_v1.3$ (*CACNA1D*) function in a human channelopathy with bradycardia and congenital deafness. *Nat. Neurosci.* **14**, 77–84 (2011).

7. A. Pinggera, A. Lieb, B. Benedetti, M. Lampert, S. Monteleone, K. R. Liedl, P. Tuluc, J. Striessnig, *CACNA1D* de novo mutations in autism spectrum disorders activate $Ca_v1.3$ L-type calcium channels. *Biol. Psychiatry* **77**, 816–822 (2015).

8. T. Frank, D. Khimich, A. Neef, T. Moser, Mechanisms contributing to synaptic Ca^{2+} signals and their heterogeneity in hair cells. *Proc. Natl. Acad. Sci. U.S.A.* **106**, 4483–4488 (2009).

9. A. C. Meyer, T. Frank, D. Khimich, G. Hoch, D. Riedel, N. M. Chapochnikov, Y. M. Yarin, B. Harke, S. W. Hell, A. Egner, T. Moser, Tuning of synapse number, structure and function in the cochlea. *Nat. Neurosci.* **12**, 444–453 (2009).

10. N. Y. S. Kiang, T. Watanabe, E. C. Thomas, L. F. Clark, *Discharge Patterns of Single Fibers in the Cat's Auditory Nerve* (MIT Press, 1965).

11. W. S. Rhode, P. H. Smith, Characteristics of tone-pip response patterns in relationship to spontaneous rate in cat auditory nerve fibers. *Hear. Res.* **18**, 159–168 (1985).

12. I. M. Winter, D. Robertson, G. K. Yates, Diversity of characteristic frequency rate-intensity functions in guinea pig auditory nerve fibres. *Hear. Res.* **45**, 191–202 (1990).

13. A. M. Taberner, M. C. Liberman, Response properties of single auditory nerve fibers in the mouse. *J. Neurophysiol.* **93**, 557–569 (2005).

14. D. Robertson, B. Paki, Role of L-type Ca^{2+} channels in transmitter release from mammalian inner hair cells. II. Single-neuron activity. *J. Neurophysiol.* **87**, 2734–2740 (2002).

15. W. F. Sewell, The relation between the endocochlear potential and spontaneous activity in auditory nerve fibres of the cat. *J. Physiol.* **347**, 685–696 (1984).

16. S. L. Johnson, Membrane properties specialize mammalian inner hair cells for frequency or intensity encoding. *eLife* **4**, e08177 (2015).

17. T.-L. Ohn, M. A. Rutherford, Z. Jing, S. Jung, C. J. Duque-Afonso, G. Hoch, M. M. Picher, A. Scharinger, N. Strenzke, T. Moser, Hair cells use active zones with different voltage dependence of Ca^{2+} influx to decompose sounds into complementary neural codes. *Proc. Natl. Acad. Sci. U.S.A.* **113**, E4716–E4725 (2016).

18. Ö. D. Özçete, T. Moser, A sensory cell diversifies its output by varying Ca^{2+} influx-release coupling among active zones. *EMBO J.* **40**, e106010 (2021).

19. A. V. Kantardzhieva, M. C. Liberman, W. F. Sewell, Quantitative analysis of ribbons, vesicles, and cisterns at the cat inner hair cell synapse: Correlations with spontaneous rate. *J. Comp. Neurol.* **521**, 3260–3271 (2013).

20. A. Merchan-Perez, M. C. Liberman, Ultrastructural differences among afferent synapses on cochlear hair cells: Correlations with spontaneous discharge rate. *J. Comp. Neurol.* **371**, 208–221 (1996).

21. S. Michanski, K. Smaluch, A. M. Steyer, R. Chakrabarti, C. Setz, D. Oestreicher, C. Fischer, W. Möbius, T. Moser, C. Vogl, C. Wichmann, Mapping developmental maturation of inner hair cell ribbon synapses in the apical mouse cochlea. *Proc. Natl. Acad. Sci. U.S.A.* **116**, 6415–6424 (2019).

22. Y. Hua, X. Ding, H. Wang, F. Wang, Y. Lu, J. Neef, Y. Gao, T. Moser, H. Wu, Electron microscopic reconstruction of neural circuitry in the cochlea. *Cell Rep.* **34**, 108551 (2021).

23. L. Grant, E. Yi, E. Glowatzki, Two modes of release shape the postsynaptic response at the inner hair cell ribbon synapse. *J. Neurosci.* **30**, 4210–4220 (2010).

24. M. B. Sachs, P. J. Abbas, Rate versus level functions for auditory-nerve fibers in cats: Tone-burst stimuli. *J. Acoust. Soc. Am.* **56**, 1835–1847 (1974).

25. M. C. Liberman, Auditory-nerve response from cats raised in a low-noise chamber. *J. Acoust. Soc. Am.* **63**, 442–455 (1978).

26. C. L. Adamson, M. A. Reid, R. L. Davis, Opposite actions of brain-derived neurotrophic factor and neurotrophin-3 on firing features and ion channel composition of murine spiral ganglion neurons. *J. Neurosci.* **22**, 1385–1396 (2002).

27. A. L. Markowitz, R. Kalluri, Gradients in the biophysical properties of neonatal auditory neurons align with synaptic contact position and the intensity coding map of inner hair cells. *eLife* **9**, e55378 (2020).

28. B. R. Shrestha, C. Chia, L. Wu, S. G. Kujawa, M. C. Liberman, L. V. Goodrich, Sensory neuron diversity in the inner ear is shaped by activity. *Cell* **174**, 1229–1246.e17 (2018).

29. S. Sun, T. Babola, G. Pregernig, K. S. So, M. Nguyen, S.-S. M. Su, A. T. Palermo, D. E. Bergles, J. C. Burns, U. Müller, Hair cell mechanotransduction regulates spontaneous activity

and spiral ganglion subtype specification in the auditory system. *Cell* **174**, 1247–1263.e15 (2018).

30. C. Petitpré, H. Wu, A. Sharma, A. Tokarska, P. Fontanet, Y. Wang, F. Helmbacher, K. Yackle, G. Silberberg, S. Hadjab, F. Lallemend, Neuronal heterogeneity and stereotyped connectivity in the auditory afferent system. *Nat. Commun.* **9**, 3691 (2018).

31. C. Li, X. Li, Z. Bi, K. Sugino, G. Wang, T. Zhu, Z. Liu, Comprehensive transcriptome analysis of cochlear spiral ganglion neurons at multiple ages. *eLife* **9**, e50491 (2020).

32. M. C. Liberman, Single-neuron labeling in the cat auditory nerve. *Science* **216**, 1239–1241 (1982).

33. H. E. Sherrill, P. Jean, E. C. Driver, T. R. Sanders, T. S. Fitzgerald, T. Moser, M. W. Kelley, Pou4f1 defines a subgroup of type I spiral ganglion neurons and is necessary for normal inner hair cell presynaptic Ca^{2+} signaling. *J. Neurosci.* **39**, 5284–5298 (2019).

34. C. Siebald, P. F. Y. Vincent, R. T. Bottom, S. Sun, D. O. J. Reijntjes, M. Manca, E. Glowatzki, U. Müller, Molecular signatures define subtypes of auditory afferents with distinct peripheral projection patterns and physiological properties. *Proc. Natl. Acad. Sci. U.S.A.* **120**, e2217033120 (2023).

35. L. M. Jaime Tobón, T. Moser, Bridging the gap between presynaptic hair cell function and neural sound encoding. *eLife* **12**, RP93749 (2024).

36. N. J. Ortner, A. Sah, E. Paradiso, J. Shin, S. Stojanovic, N. Hammer, M. Haritonova, N. T. Hofer, A. Marcantoni, L. Guarina, P. Tuluc, T. Theiner, F. Pitterl, K. Ebner, H. Oberacher, E. Carbone, N. Stefanova, F. Ferraguti, N. Singewald, J. Roeper, J. Striessnig, The human channel gating–modifying A749G *CACNA1D* ($Ca_v1.3$) variant induces a neurodevelopmental syndrome–like phenotype in mice. *JCI Insight* **8**, e162100 (2023).

37. N. J. Ortner, T. Kaserer, J. N. Copeland, J. Striessnig, De novo CACAN1D Ca^{2+} channelopathies: Clinical phenotypes and molecular mechanism. *Pflugers Arch. Eur. J. Physiol.* **472**, 755–773 (2020).

38. F. Dannenberg, A. Von Moers, P. Bittigau, J. Lange, S. Wiegand, F. Török, G. Stölting, J. Striessnig, M. M. Motazacker, M. F. Broekema, M. Schuelke, A. M. Kaindl, U. I. Scholl, N. J. Ortner, A novel de novo gain-of-function *CACNA1D* variant in neurodevelopmental disease with congenital tremor, seizures, and hypotonia. *Neurol. Genet.* **10**, e200186 (2024).

39. D. Oestreicher, S. Chepurwar, K. Kusch, V. Rankovic, S. Jung, N. Strenzke, T. Pangrsic, CaBP1 and 2 enable sustained $Ca_V1.3$ calcium currents and synaptic transmission in inner hair cells. *eLife* **13**, RP93646 (2024).

40. M. M. Picher, A. Gehrt, S. Meese, A. Ivanovic, F. Predoehl, S. Jung, I. Schrauwen, A. G. Dragonetti, R. Colombo, G. V. Camp, N. Strenzke, T. Moser, Ca^{2+}-binding protein 2 inhibits Ca^{2+}-channel inactivation in mouse inner hair cells. *Proc. Natl. Acad. Sci. U.S.A.* **114**, E1717–E1726 (2017).

41. I. Schrauwen, S. Helfmann, A. Inagaki, F. Predoehl, M. A. Tabatabaiefar, M. M. Picher, M. Sommen, C. Z. Seco, J. Oostrik, H. Kremer, A. Dheedene, C. Claes, E. Fransen, M. H. Chaleshtori, P. Coucke, A. Lee, T. Moser, G. Van Camp, A mutation in CABP2, expressed in cochlear hair cells, causes autosomal-recessive hearing impairment. *Am. J. Hum. Genet.* **91**, 636–645 (2012).

42. S. G. Kujawa, M. C. Liberman, Adding insult to injury: Cochlear nerve degeneration after "temporary" noise-induced hearing loss. *J. Neurosci.* **29**, 14077–14085 (2009).

43. A. C. Furman, S. G. Kujawa, M. C. Liberman, Noise-induced cochlear neuropathy is selective for fibers with low spontaneous rates. *J. Neurophysiol.* **110**, 577–586 (2013).

44. Y. Lu, J. Liu, B. Li, H. Wang, F. Wang, S. Wang, H. Wu, H. Han, Y. Hua, Spatial patterns of noise-induced inner hair cell ribbon loss in the mouse mid-cochlea. *iScience* **27**, 108825 (2024).

45. P. Hess, J. B. Lansman, R. W. Tsien, Different modes of Ca channel gating behaviour favoured by dihydropyridine Ca agonists and antagonists. *Nature* **311**, 538–544 (1984).

46. W. M. Roberts, R. A. Jacobs, A. J. Hudspeth, Colocalization of ion channels involved in frequency selectivity and synaptic transmission at presynaptic active zones of hair cells. *J. Neurosci.* **10**, 3664–3684 (1990).

47. A. Brandt, D. Khimich, T. Moser, Few $Ca_V1.3$ channels regulate the exocytosis of a synaptic vesicle at the hair cell ribbon synapse. *J. Neurosci.* **25**, 11577–11585 (2005).

48. D. Zenisek, N. K. Horst, C. Merrifield, P. Sterling, G. Matthews, Visualizing synaptic ribbons in the living cell. *J. Neurosci.* **24**, 9752–9759 (2004).

49. J. Neef, N. T. Urban, T.-L. Ohn, T. Frank, P. Jean, S. W. Hell, K. I. Willig, T. Moser, Quantitative optical nanophysiology of Ca^{2+} signaling at inner hair cell active zones. *Nat. Commun.* **9**, 290 (2018).

50. J. S. Marvin, B. G. Borghuis, L. Tian, J. Cichon, M. T. Harnett, J. Akerboom, A. Gordus, S. L. Renninger, T.-W. Chen, C. I. Bargmann, M. B. Orger, E. R. Schreiter, J. B. Demb, W.-B. Gan, S. A. Hires, L. L. Looger, An optimized fluorescent probe for visualizing glutamate neurotransmission. *Nat. Methods* **10**, 162–170 (2013).

51. F. K. Wong, A. R. Nath, R. H. C. Chen, S. R. Gardezi, Q. Li, E. F. Stanley, Synaptic vesicle tethering and the $Ca_V2.2$ distal C-terminal. *Front. Cell. Neurosci.* **8**, 71 (2014).

52. T. Pangršič, M. Gabrielaitis, S. Michanski, B. Schwaller, F. Wolf, N. Strenzke, T. Moser, EF-hand protein Ca^{2+} buffers regulate Ca^{2+} influx and exocytosis in sensory hair cells. *Proc. Natl. Acad. Sci. U.S.A.* **112**, E1028–E1037 (2015).

53. Z. Jing, M. A. Rutherford, H. Takago, T. Frank, A. Fejtova, D. Khimich, T. Moser, N. Strenzke, Disruption of the presynaptic cytomatrix protein bassoon degrades ribbon anchorage, multiquantal release, and sound encoding at the hair cell afferent synapse. *J. Neurosci.* **33**, 4456–4467 (2013).

54. R. E. Study, Isoflurane inhibits multiple voltage-gated calcium currents in hippocampal pyramidal neurons. *Anesthesiology* **81**, 104–116 (1994).

55. F. Török, K. Tezcan, L. Filippini, M. L. Fernández-Quintero, L. Zanetti, K. R. Liedl, R. S. Drexel, J. Striessnig, N. J. Ortner, Germline de novo variant F747S extends the phenotypic spectrum of *CACNA1D* Ca^{2+} channelopathies. *Hum. Mol. Genet.* **32**, 847–859 (2023).

56. D. Oestreicher, A. M. Malpede, A. Reitmeier, C. P. Bräuer, L. Schoch, N. Strenzke, T. Pangrsic, Noise-induced ribbon synapse loss in the mouse basal cochlear region does not reduce inner hair cell exocytosis. *Front. Cell. Neurosci.* **18**, 1523978 (2024).

57. C. J. Doering, G. W. Zamponi, Molecular pharmacology of high voltage-activated calcium channels. *J. Bioenerg. Biomembr.* **35**, 491–505 (2003).

58. R. Meddis, M. J. Hewitt, T. M. Shackleton, Implementation details of a computation model of the inner hair-cell auditory-nerve synapse. *J. Acoust. Soc. Am.* **87**, 1813–1816 (1990).

59. A. B. Wong, Z. Jing, M. A. Rutherford, T. Frank, N. Strenzke, T. Moser, Concurrent maturation of inner hair cell synaptic Ca^{2+} influx and auditory nerve spontaneous activity around hearing onset in mice. *J. Neurosci.* **33**, 10661–10666 (2013).

60. L. Robles, M. A. Ruggero, Mechanics of the mammalian cochlea. *Physiol. Rev.* **81**, 1305–1352 (2001).

61. B. Pan, G. S. Geleoc, Y. Asai, G. C. Horwitz, K. Kurima, K. Ishikawa, Y. Kawashima, A. J. Griffith, J. R. Holt, TMC1 and TMC2 are components of the mechanotransduction channel in hair cells of the mammalian inner ear. *Neuron* **79**, 504–515 (2013).

62. H.-T. C. Wong, Q. Zhang, A. J. Beirl, R. S. Petralia, Y.-X. Wang, K. Kindt, Synaptic mitochondria regulate hair-cell synapse size and function. *eLife* **8**, e48914 (2019).

63. L. Sheets, K. S. Kindt, T. Nicolson, Presynaptic $Ca_V1.3$ channels regulate synaptic ribbon size and are required for synaptic maintenance in sensory hair cells. *J. Neurosci.* **32**, 17273–17286 (2012).

64. M. Lindau, E. Neher, Patch-clamp techniques for time-resolved capacitance measurements in single cells. *Pflügers Arch. Eur. J. Physiol.* **411**, 137–146 (1988).

65. T. Moser, D. Beutner, Kinetics of exocytosis and endocytosis at the cochlear inner hair cell afferent synapse of the mouse. *Proc. Natl. Acad. Sci. U.S.A.* **97**, 883–888 (2000).

66. J. Neef, A. Gehrt, A. V. Bulankina, A. C. Meyer, D. Riedel, R. G. Gregg, N. Strenzke, T. Moser, The Ca^{2+} channel subunit β2 regulates Ca^{2+} channel abundance and function in inner hair cells and is required for hearing. *J. Neurosci.* **29**, 10730–10740 (2009).

67. K. N. Richter, N. H. Revelo, K. J. Seitz, M. S. Helm, D. Sarkar, R. S. Saleeb, E. D'Este, J. Eberle, E. Wagner, C. Vogl, D. F. Lazaro, F. Richter, J. Coy-Vergara, G. Coceano, E. S. Boyden, R. R. Duncan, S. W. Hell, M. A. Lauterbach, S. E. Lehnart, T. Moser, T. Outeiro, P. Rehling, B. Schwappach, I. Testa, B. Zapiec, S. O. Rizzoli, Glyoxal as an alternative fixative to formaldehyde in immunostaining and super-resolution microscopy. *EMBO J.* **37**, 139–159 (2018).

68. A. B. Wong, M. A. Rutherford, M. Gabrielaitis, T. Pangršič, F. Göttfert, T. Frank, S. Michanski, S. Hell, F. Wolf, C. Wichmann, T. Moser, Developmental refinement of hair cell synapses tightens the coupling of Ca^{2+} influx to exocytosis. *EMBO J.* **33**, 247–264 (2014).

69. P. Jean, D. L. de la Morena, S. Michanski, L. M. J. Tobón, R. Chakrabarti, M. M. Picher, J. Neef, S. Jung, M. Gültas, S. Maxeiner, A. Neef, C. Wichmann, N. Strenzke, C. Grabner, T. Moser, The synaptic ribbon is critical for sound encoding at high rates and with temporal precision. *eLife* **7**, e29275 (2018).

70. Y. Hua, S. Loomba, V. Pawlak, K.-M. Voit, P. Laserstein, K. M. Boergens, D. J. Wallace, J. N. D. Kerr, M. Helmstaedter, Connectomic analysis of thalamus-driven disinhibition in cortical layer 4. *Cell Rep.* **41**, 111476 (2022).

71. Y. Lu, Y. Jiang, F. Wang, H. Wu, Y. Hua, Electron microscopic mapping of mitochondrial morphology in the cochlear nerve fibers. *JARO* **25**, 341–354 (2024).

72. P. Jean, Ö. D. Özçete, B. Tarchini, T. Moser, Intrinsic planar polarity mechanisms influence the position-dependent regulation of synapse properties in inner hair cells. *Proc. Natl. Acad. Sci. U.S.A.* **116**, 9084–9093 (2019).

73. N. Karagulyan, T. Moser, Synaptic activity is not required for establishing heterogeneity of inner hair cell ribbon synapses. *Front. Mol. Neurosci.* **16**, 1248941 (2023).

74. M. Sanchez del Rio, G. Pareschi, "Global optimization and reflectivity data fitting for x-ray multilayer mirrors by means of genetic algorithms" in *Proceedings of SPIE—The International Society for Optical Engineering* (SPIE, 2001), vol. 4145, pp. 88–96.

75. B. N. Buran, N. Strenzke, A. Neef, E. D. Gundelfinger, T. Moser, M. C. Liberman, Onset coding is degraded in auditory nerve fibers from mutant mice lacking synaptic ribbons. *J. Neurosci.* **30**, 7587–7597 (2010).
